# An N-Cadherin 2 expressing epithelial cell subpopulation predicts response to surgery, chemotherapy and immunotherapy in bladder cancer

**DOI:** 10.1038/s41467-021-25103-7

**Published:** 2021-08-12

**Authors:** Kenneth H. Gouin, Nathan Ing, Jasmine T. Plummer, Charles J. Rosser, Bassem Ben Cheikh, Catherine Oh, Stephanie S. Chen, Keith Syson Chan, Hideki Furuya, Warren G. Tourtellotte, Simon R. V. Knott, Dan Theodorescu

**Affiliations:** 1 Department of Biomedical Sciences, Cedars-Sinai Medical Center, Los Angeles, CA USA; 2 Center for Bioinformatics and Functional Genomics, Cedars-Sinai Medical Center, Los Angeles, CA USA; 3 Department of Surgery (Urology), Cedars-Sinai Medical Center, Los Angeles, CA USA; 4 Cedars-Sinai Samuel Oschin Comprehensive Cancer Institute, Los Angeles, CA USA; 5 Department of Pathology and Laboratory Medicine, Cedars-Sinai Medical Center, Los Angeles, CA USA; 6 Department of Neurology, Cedars-Sinai Medical Center, Los Angeles, CA USA; 7 Department of Neurosurgery, Cedars-Sinai Medical Center, Los Angeles, CA USA

**Keywords:** Bladder cancer, Mechanisms of disease, Predictive markers

## Abstract

Neoadjuvant chemotherapy (NAC) prior to surgery and immune checkpoint therapy (ICT) have revolutionized bladder cancer management. However, stratification of patients that would benefit most from these modalities remains a major clinical challenge. Here, we combine single nuclei RNA sequencing with spatial transcriptomics and single-cell resolution spatial proteomic analysis of human bladder cancer to identify an epithelial subpopulation with therapeutic response prediction ability. These cells express *Cadherin 12* (*CDH12, N-Cadherin 2*), catenins, and other epithelial markers. CDH12-enriched tumors define patients with poor outcome following surgery with or without NAC. In contrast, CDH12-enriched tumors exhibit superior response to ICT. In all settings, patient stratification by tumor CDH12 enrichment offers better prediction of outcome than currently established bladder cancer subtypes. Molecularly, the CDH12 population resembles an undifferentiated state with inherently aggressive biology including chemoresistance, likely mediated through progenitor-like gene expression and fibroblast activation. CDH12-enriched cells express PD-L1 and PD-L2 and co-localize with exhausted T-cells, possibly mediated through CD49a (*ITGA1*), providing one explanation for ICT efficacy in these tumors. Altogether, this study describes a cancer cell population with an intriguing diametric response to major bladder cancer therapeutics. Importantly, it also provides a compelling framework for designing biomarker-guided clinical trials.

## Abstract (web summary)

The identification of response biomarkers for surgery, chemotherapy and immune checkpoint therapy in bladder cancer is crucial. Here, single nuclei RNA sequencing and spatial profiling identify a cancer cell population expressing Neural Type Cadherin 2 that associates with distinct treatment outcomes.

## Introduction

Molecular subtyping of muscle-invasive bladder cancer (MIBC) has revolutionized the current conceptual thinking of MIBC pathogenesis^1–6^. However, even the most recent consensus molecular classification systems do not provide compelling evidence for its use in clinical decision-making and is specifically lacking in predictions for therapeutic response. Emerging studies using single-cell RNA-sequencing to analyze MIBC have provided an initial understanding of intratumoral heterogeneity^7^. However, these studies have focused on the tumor microenvironment, have been limited by relatively small cohort sizes, and have yet to provide a clearer path toward therapeutic decision-making. We hypothesized that comprehensive profiling at the single-cell level of MIBC epithelial and non-epithelial cells would serve to deconvolute current molecular subtypes into their constituent parts and consequently develop more effective prognostic and predictive tools.

In this study, we perform the first comprehensive profiling of high-grade urothelial MIBCs using single-nucleus RNA-sequencing (snSeq) on 25 treatment-naïve patients, with surgery (TURBT/cystectomy) as their only treatment. We demonstrate the presence of a previously uncharacterized epithelial cell phenotype marked by high expression of Cadherin 12 (*CDH12*, N-Cadherin 2), catenins and other epithelial markers. We further show that this phenotype is present in multiple established molecular subtypes, demonstrating intra-subtype heterogeneity. We also find that CDH12-enriched tumors define patients with poor outcome following surgery with or without neoadjuvant chemotherapy, but superior outcome in the context of immune checkpoint therapy (ICT). Finally, using in-situ profiling we demonstrate that CDH12-enriched epithelial cells reside in distinct cellular niches that are enriched for exhausted CD8 T-cells, thus elucidating a possible mechanistic explanation for their ability to predict response to ICT.

## Results

### Identification of stem-like *CDH12*-expressing epithelial cells

**Fig. 1:**
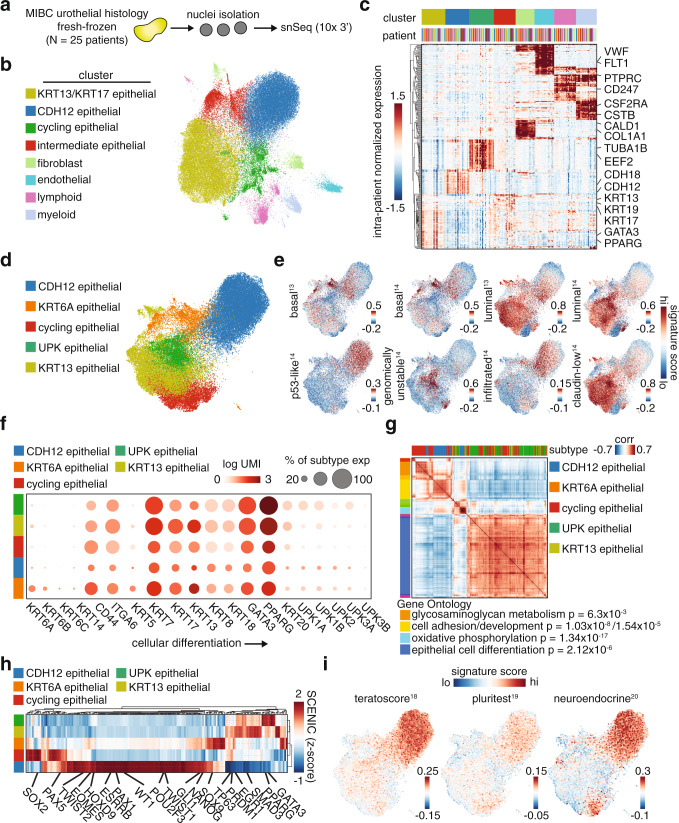
Discovery of a CDH12+ tumor cell population by single-nucleus sequencing. **a** Workflow for single nucleus sequencing; MIBC—muscle invasive bladder cancer. **b** UMAP of all nuclei (71,832) in MIBC dataset colored by unsupervised clustering. **c** Average gene expression per patient of marker genes for each cell type in **b**. **d** UMAP of all epithelial nuclei (52,983) in MIBC dataset colored by epithelial population. **e** Gene signature scores for published MIBC subtype gene sets. **f** Uroepithelial differentiation-related marker gene expression in each epithelial population, where the dot size indicates the percent of cells within the subtype with non-zero expression of the respective gene. **g** Gene-gene correlations partitioned into co-expression modules annotated for epithelial population enrichment. Gene ontology (GO) annotations are included with g:SCS multiple testing corrected p-values for hypergeometric testing. **h** Activity scores for SCENIC transcription factor regulons in each epithelial population. **i** Gene signature scores for stem-cell and neuroendocrine differentiation gene sets.

Toward the first goal of characterizing intratumoral heterogeneity, we first looked at the overall cellular composition of the profiled MIBC tumors based on snSeq cell type proportions (Fig. 1a, Supplementary Fig. 1a, b, and Supplementary Table 1). The tumors were composed of ~90% epithelial cells, 5% immune cells, 3% fibroblasts, and 2% endothelial cells as annotated based on their corresponding expression of keratins, *PTPRC*, collagens, *PECAM1*, and *VWF*, respectively, among other key marker genes (Fig. 1b, c and Supplementary Fig. 1c). Unsupervised clustering of the epithelial compartment alone identified clusters with differential expression of *KRT13* and *KRT17*, which were combined into one cluster (KRT13), uroplakins (UPK), *KRT6A*, cell-cycle-related genes (cycling), as well as a distinct cellular population expressing *CDH12* along with other epithelial markers (Fig. 1d and Supplementary Fig. 1e). We observed substantial inter-tumoral heterogeneity in epithelial compositions (Supplementary Fig. 1f). The aforementioned genes were used to annotate the clusters because their high expression denoted unique clusters and the genes hold functional relevance (the complete listing of differentially expressed genes for each cluster can be found in Supplementary Data File 1). The fibroblasts encompassed 4 major populations defined by key cancer-associated fibroblast (CAF) markers^8–11^, including fibroblast activation protein (*FAP*), alpha smooth muscle actin (αSMA, *ACTA2*), podoplanin (*PDPN*), and platelet-derived growth factor receptor beta (*PDGFRβ*) (Supplementary Fig. 1g, h). The immune compartment contained a diverse collection of cells including T-cells, dendritic cells, macrophages, and B-cells as defined by classic immune marker genes (Supplementary Fig. 1i, j).

We focused on a deeper analysis of the epithelial compartment as it constituted the bulk of the tumor. Immunohistochemistry verified the expression of KRT13, KRT17, and CDH12 in tumors that were predicted by snSeq to have high versus low levels of KRT13 and CDH12 epithelial populations (Supplementary Figs. 2 and 3). We then evaluated the epithelial populations in the context of previously published MIBC gene signatures to determine similarities and differences. The KRT13 and UPK populations were most closely related to the luminal phenotype, while the KRT6A population was similar to the basal phenotype. Interestingly, the CDH12 population had elements of the p53-like and immune-infiltrated phenotypes suggesting that it may be present to some degree in multiple previously established subtypes, and that prior methods were unable to fully elucidate its molecular contribution to MIBC^12,13^. The KRT13 and UPK populations were the only two that lacked the gene signature derived from immune-infiltrated MIBC, suggesting that tumors that are enriched for these populations represent immunologically “cold” tumors (Fig. 1e)^14^. Further cross-referencing to conventional uroepithelial differentiation-related markers indicated that the KRT13 and UPK populations represented a more differentiated phenotype, while the CDH12, KRT6A, and cycling populations represented an undifferentiated or dedifferentiated phenotype (Fig. 1f).

To further characterize the epithelial populations, we performed several unbiased analyses. We constructed a gene network consisting of variably expressed genes with high pair-wise correlations, and used gene ontology enrichment to understand the function of the resultant subnetworks. As expected, the KRT13 and UPK populations expressed an epithelial cell differentiation network (Fig. 1g). Further underscoring the unique nature of the CDH12 population, we found these cells express cell adhesion and cell development pathways. Gene expression scoring for the identified subnetworks showed significant enrichment in the corresponding epithelial populations as expected (Supplementary Fig. 4a). The CDH12 population was also predicted to exhibit high activity of several development-related transcription factors based on Single-Cell rEgulatory Network Inference and Clustering (SCENIC) analysis, including NANOG, EOMES, PAX1, and HOXD9 (Fig. 1h). In contrast, the UPK and KRT13 populations exhibited higher activity of the differentiation regulators PPARG and GATA3^15^. The CDH12 and cycling populations also scored highly for stem-like (teratoscore/pluritest) and neuroendocrine gene signatures (Fig. 1i)^16–18^. Consistent with a stem-like phenotype, we also found that the CDH12 population differentially expressed *ALDH1A1*, a key bladder stem cell marker (Supplementary Fig. 4b)^19^.

### CDH12-enriched cells are found in healthy, normal bladder epithelium

**Fig. 2:**
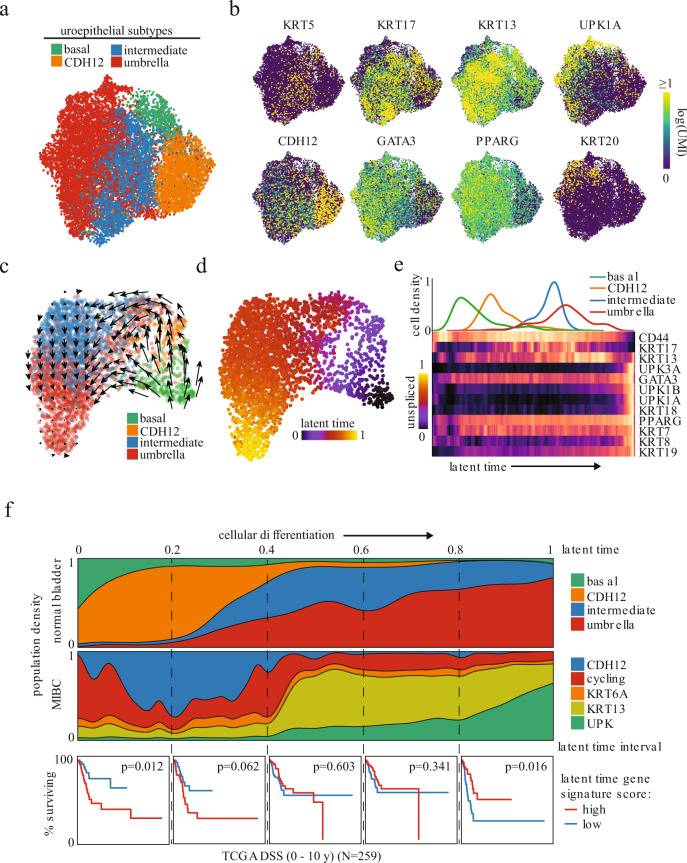
CDH12+ tumor population resembles characteristics of early undifferentiated urothelial cells and correlates with poor clinical outcome. **a** UMAP of 12,819 uroepithelial nuclei obtained from histologically normal bladder and colored by unsupervised clustering. **b** Uroepithelial differentiation-related marker gene expression. **c** RNA velocity latent time trajectory in healthy bladder epithelial nuclei from a representative patient. **d** RNA velocity-based latent time of the nuclei shown in **c**. **e** Epithelial population density (top) and heatmap of uroepithelial marker gene expression (bottom) in nuclei from **d** ordered by increasing latent time. **f** Epithelial population distribution across latent time for all normal samples combined (top row) or MIBC samples based on normal nearest neighbor analysis (middle row). Normal samples were combined by collating the latent times from velocity analyses performed on each of the 4 samples independently. Disease-specific survival of high-grade MIBC in TCGA stratified by gene signature scores derived from MIBC nuclei in the latent time intervals demarcated by the dashed lines (bottom row, log-rank test between top and bottom quartiles *N* = 259).

To gain insights into the biological origin and differentiation path of the newly identified epithelial populations, we also performed snSeq profiling on 4 histologically normal bladder samples. Unsupervised clustering of the epithelial cells identified basal, intermediate, and umbrella populations, as previously described (Fig. 2a and Supplementary Fig. 4c)^20^. Interestingly, the CDH12 population was clearly distinct from these latter canonical groups, while the intermediate cells expressed the highest levels of *KRT13* and *KRT17* (Fig. 2b). In addition, the CDH12 population from these samples expressed lower levels of genes known to be amplified in bladder cancer compared to their MIBC counterpart, including *TERT* and *SOX4* (Supplementary Fig. 4d)^21^. We applied RNA velocity analysis to each sample individually, using information about the expression of genes at the unspliced and spliced level to predict a pseudotime trajectory^22,23^. This identified a trajectory that initiated in basal cells and subsequently diverged into two differentiation paths: one traveling through the CDH12 population and one that skips the CDH12 population (Fig. 2c, d, representative sample shown). Both paths ultimately converge on the intermediate population and terminate in the umbrella population. Key uroepithelial differentiation markers tracked along this path as expected, with high expression of *CD44* at initiation, followed by *KRT13* and *KRT17* in the middle, and *UPK1A*, *GATA3*, and *PPARG* at the terminus (Fig. 2e). Pseudotime trajectories of all 4 normal samples exhibited similar paths, with the CDH12 population situated near the initiation (Fig. 2f, top and Supplementary Fig. 4e). Taken together, this demonstrated that the CDH12 population was a distinct node in the path of bladder differentiation. Conceivably, transformation at this juncture would lead to tumor development with an enrichment of the CDH12 population.

To determine the transcriptional similarity between the CDH12 tumor cells and their normal counterparts and infer their position along the normal epithelial differentiation trajectory, we identified the nearest normal cell neighbor of every MIBC epithelial cell, using expression similarities, and then assigned the corresponding normal latent times to the tumor cells (Fig. 2f, middle). This revealed that the CDH12, cycling, and KRT6A populations were most consistent with an undifferentiated or dedifferentiated phenotype, while the UPK population was most consistent with a fully differentiated phenotype. We then sought to understand the predictive potential of this trajectory as previous studies had identified luminal (differentiated) and basal (undifferentiated) signatures as prognostically relevant^24,25^. We created gene signatures from intervals along our identified differentiation paths and scored 259 samples of previously untreated high-grade urothelial MIBC tumors in The Cancer Genome Atlas (TCGA, Supplementary Data File 2) for each interval using single-sample gene set enrichment analysis (ssGSEA)^26^. Strikingly, the interval score corresponding to the most undifferentiated phenotype predicted poor disease-specific survival (DSS) while the interval score of the most differentiated phenotype predicted better DSS, with the interval scores in between demonstrating a transition between the opposing outcomes (Fig. 2f, bottom).

### CDH12 score predicts poor prognosis in MIBC

**Fig. 3:**
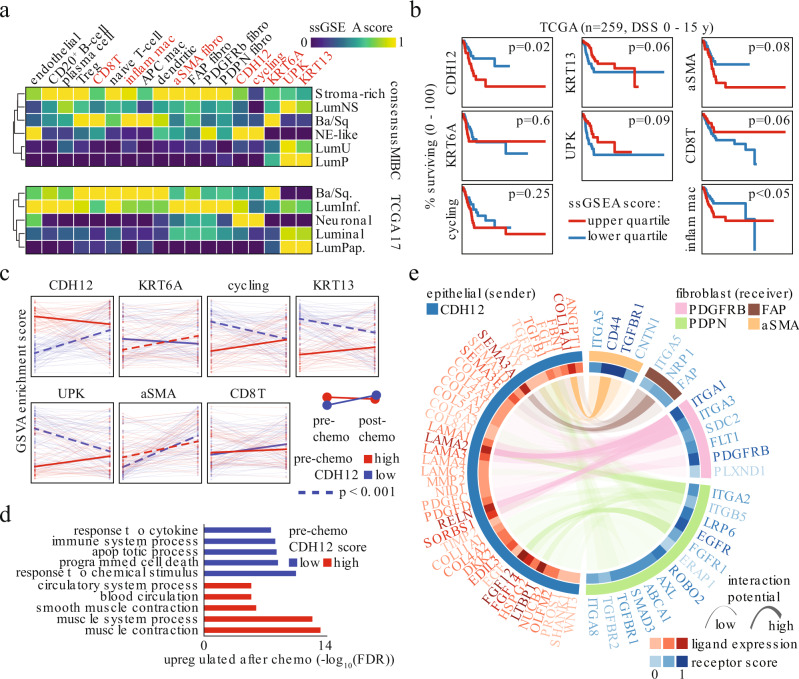
High CDH12 scores predict chemoresistance and fibroblast activation. **a** Average snSeq-derived signature scores in molecular subtypes of TCGA MIBC cases (*N* = 259). Signatures highlighted in orange are shown in **b**. **b** Disease specific survival of high-grade MIBC in TCGA stratified by snSeq population signatures (log-rank test between top and bottom quartiles, *N* = 259). **c** Tracking of 7 snSeq population signature scores in matched pre-chemo (left edge) and post-chemo samples (right edge) stratified by their pre-chemo CDH12 signature score (dark line indicates median of all samples shown as light lines, blue lines—low pre-chemo CDH12 score, red lines—high pre-chemo CDH12 score) (dashed line indicates *p* < 0.001 for post-versus pre-chemo scores, Wilcoxon paired one-sided rank-sum test). **d** GO term enrichment (hypergeometric overlap test) for genes up-regulated post-chemo in tumors with low or high CDH12 score in the pre-chemo setting. **e** snSeq-derived receptor-ligand interactions significantly enriched between the CDH12 population and each fibroblast population (see Methods for details).

The observed prognostic value of the differentiation path gene signatures and their relationship to the CDH12 population prompted us to delve further into analyzing TCGA high-grade MIBC tumors. We created gene signatures for each of our cellular populations (Supplementary Data File 3) and scored each TCGA sample for these signatures using ssGSEA. We created cellular profiles for each of the TCGA tumors and analyzed them in the context of the consensus MIBC or TCGA 2017 classifications (Fig. 3a)^21,27^. Not surprisingly, we observed good agreement between classification systems. Our UPK signature was enriched in the luminal subtypes, while our KRT6A signature was enriched in the basal/squamous (Ba/Sq) subtypes. Interestingly, speaking to its unique nature, the CDH12 signature distributed across the Ba/Sq, luminal infiltrated, and neuroendocrine-like subtypes, while being notably absent from the luminal papillary (LumP) and luminal uncertain (LumU) subtypes (Fig. 3a). This was consistent with our previous observation that the CDH12 population may be present to some degree in multiple previously established subtypes (Fig. 1e). The Ba/Sq and luminal infiltrated subtypes, which harbored CDH12 enrichment, also demonstrated enrichment for CD8^+^ T-cells and fibroblasts, which was notably lacking in the LumP and LumU subtypes. The CDH12 and macrophage signatures were the lone predictors of poor DSS (Fig. 3b). Notably, the KRT13, UPK, and CD8^+^ T-cell (CD8T) signatures were linked with better DSS and αSMA fibroblasts with poorer DSS, however these associations did not reach the level of statistical significance.

### CDH12 score predicts poor response to neoadjuvant chemotherapy

Having established the broad prognostic impact of our molecular signatures on surgically treated MIBC, we investigated their ability to predict response to platinum-based chemotherapy using data from paired pre- and post-NAC bladder cancer samples from a recent study^13,28^. Our gene signatures tracked with the single-sample classifier reported in the study in a manner consistent with the TCGA subtyping (Supplementary Fig. 5a). While our gene signatures did not predict response rate based on pathological downstaging (Supplementary Fig. 5b), once again the CDH12 score predicted poor overall survival (OS), while the KRT13 and UPK (*p* = 0.06) scores predicted better OS (Supplementary Fig. 5c). To determine how the CDH12 population might associate with changes brought about by chemotherapy, we split pre-chemotherapy samples by high and low CDH12 scores and tracked changes in our gene signatures following chemotherapy. We observed low CDH12 score samples tended to become high CDH12 score samples after chemotherapy, while high CDH12 score samples tended to retain a high CDH12 score after chemotherapy (Fig. 3c). In contrast, the opposite trend was observed when performing a similar analysis using the UPK signature score, while the other epithelial populations did not exhibit any clear progression. This suggests that the CDH12 population is chemo-resistant, while the UPK population is chemo-sensitive. Interestingly, both tumor types increased in αSMA score after chemotherapy, indicating potential stromal activation. Tumors that started with low CD8T scores tended to increase their CD8T score after chemotherapy, indicating immune activation.

### CDH12 cells are chemo-resistant and activate stroma

To further understand the changes brought on by chemotherapy in the context of CDH12, we compared gene expression profiles of matched post-chemotherapy and pre-chemotherapy tumors separated by their pre-chemotherapy CDH12 score. Interestingly, tumors that began with a low CDH12 score increased expression of genes related to apoptosis and immune activation in response to chemotherapy, while tumors that started with a high CDH12 score responded to chemotherapy through fibroblast and endothelial cell activation (Fig. 3d). This stromal activation signature prompted us to search for potential communication between the CDH12 epithelial cells and fibroblasts in our snSeq data. Using ligand-receptor interaction analysis, see Methods for details, we looked for interactions in which the ligand was differentially expressed by the CDH12 population versus the other epithelial populations and the receiving population demonstrated differential activity of the matching receptor^29,30^. We observed many significantly enriched interactions between the CDH12 population and fibroblasts, with the most notable being TGFBR1, CD44, and several integrins because of their involvement in cancer-associated fibroblast (CAF) activation (Fig. 3e). TGFβ activates CAFs in a partially CD44-dependent manner, resulting in their proliferation and promotion of the epithelial-to-mesenchymal transition and wound-healing pathways^31–33^. Taken together, these observations suggest the CDH12 population may represent a chemo-resistant tumor subpopulation characterized by TGFβ-induced CAF activation, while the KRT13 and UPK populations represent chemo-sensitive subpopulations that may undergo apoptosis and induce immune activation through immunogenic cell death pathways^34,35^.

### CDH12 score predicts immunotherapy response post-chemotherapy

**Fig. 4:**
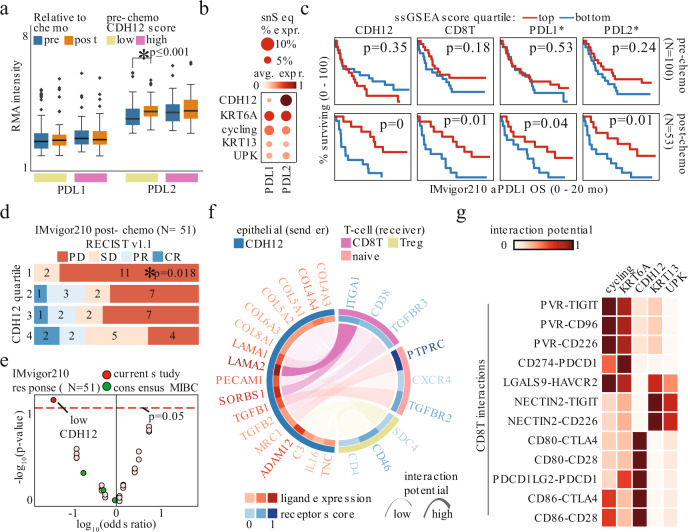
Post-chemo CDH12 score predicts favorable response to immune checkpoint therapy. **a** PDL1 and PDL2 in matched pre-chemo and post-chemo samples (* - Wilcoxon paired two-sided rank-sum test *p* < 0.05; *n* = 65 for low CDH12, *n* = 49 for high CDH12). Boxplots are drawn as the inter-quartile range (IQR) with a line indicating the median, and outliers defined as points that fall outside of the range demarcated by 1.5*IQR. Source data are available as a Source data file. **b** PDL1 and PDL2 expression in snSeq tumor epithelial cells. **c** Overall survival in IMvigor 210 Cohort 2 bladder tumors sequenced pre-chemo (top, *N* = 100) or post-chemo (bottom, *N* = 53) stratified by snSeq-derived population signature scores, or gene expression value (log-rank test, *p* = 0 indicates *p* < 0.001; * indicates gene expression). **d** RECIST v1.1 response in bladder tumors profiled post-chemo stratified by CDH12 score quartile; progressive disease (PD), stable disease (SD), partial response (PR), complete response (CR) (* - Fisher exact test for PD vs PR/CR in quartile 1 vs quartile 4, *N* = 51). **e** Association of snSeq-derived signature scores, or consensus MIBC subtypes, with RECIST v1.1 response in the IMvigor 210 Cohort 2 cases shown in **d** (Fisher exact test, *N* = 51). **f** snSeq-derived receptor-ligand interactions significantly enriched between CDH12 population and each T-cell population. **g** snSeq-derived receptor-ligand interaction potential of co-inhibitory signaling from epithelial populations to the CD8T population.

Since tumors with low baseline CDH12 scores responded to chemotherapy with a concomitant rise in their CDH12, apoptosis, and immune activation gene signatures, we also investigated the corresponding changes to immune checkpoint-related genes. With immune activation, we found tumors with low CDH12 scores increased their expression of *PDCD1LG2* (PDL2) after chemotherapy, while PDL2 expression was higher than PDL1 (*CD274*) expression in all samples (Fig. 4a). The former observation was consistent with our snSeq dataset showing CDH12 cells expressed the highest level of PDL2 among the epithelial populations (Fig. 4b). This led us to examine our gene signatures in the context of the IMvigor210 trial. This trial investigated, in what the original authors termed Cohort 2, the efficacy of the anti-PDL1 antibody atezolizumab in patients who previously failed to respond to platinum-based chemotherapy^36^. Given our observation that chemotherapy substantially alters tumor composition by enriching for the CDH12 population (Fig. 3c), we split the IMvigor210 cohort into samples originating from bladder that were taken pre-chemotherapy or post-chemotherapy (Supplementary Fig. 6a, see methods for cohort selection details). Consistent with the results of the NAC cohort, in the pre-chemotherapy samples CDH12 levels were associated with poor OS, albeit not significantly. Strikingly, however, CDH12 levels predicted better OS in the post-chemotherapy samples (Fig. 4c). Scores pertaining to the other epithelial populations as well as the αSMA population exhibited similar differential prognostic values in the pre- versus post-chemotherapy setting, i.e. predicting poor versus better OS in the pre-chemotherapy versus post-chemotherapy settings (Supplementary Fig. 6b). Furthermore, only in the post-chemotherapy setting did the CD8T score and expression of PDL1 and PDL2 demonstrate significant prognostic value (Fig. 4c). The CDH12 score was also associated with pathological response in the post-chemotherapy setting and, indeed, it was the only factor with a significant association with response in the post-chemotherapy setting, even when considering the well-established consensus MIBC subtypes (Fig. 4d, e). Altogether, this suggests that the history of the tumor is critically important for therapeutic decision-making, as the tumor composition prior to chemotherapy portends the changes that will occur in response to chemotherapy, which then informs prognosis and response for subsequent targeting of the PD1/PDL1 axis.

### CDH12 cells interact with CD8 T-cells through CD49a

To further understand how the presence of CDH12 cells impacts response to PDL1 blockade, we examined our snSeq cohort for specific ligand-receptor interactions with T-cells. While we again found numerous significant interactions between CDH12 epithelial cells and T-cells, we identified the strongest interaction to be *ITGA1*, which codes for CD49a, on CD8T (Fig. 4f). CD49a is the alpha 1 subunit of integrin receptors and heterodimerizes with the beta 1 subunit to form a cell-surface receptor for collagen and laminin. The heterodimeric receptor is involved in cell–cell adhesion, inflammation, and fibrosis^37–39^. CD49a plays a critical role in CD8T migration and surveillance of peripheral tissues. Its blockade or deletion results in impaired accumulation of CD8T in peripheral tissues, indicating that this interaction may partly explain the CD8T persistence in CDH12-high tumors^37–39^. In a targeted analysis of checkpoint interactions, we identified the CDH12 population as having the strongest PDL2-PD1 (*PDCD1LG2*-*PDCD1*) and CTLA-4 interactions with CD8T, while the KRT13 and UPK populations interacted with CD8T through TIGIT and TIM-3 (HAVCR2) (Fig. 4g).

### CDH12 cells co-localize with CD8 T-cells

**Fig. 5:**
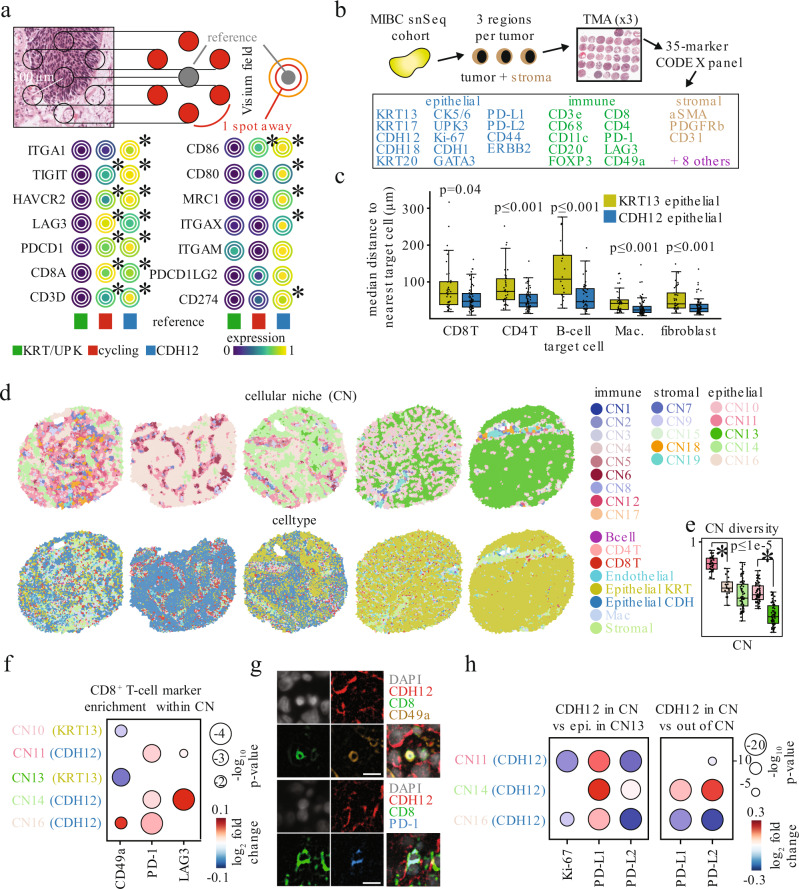
CDH12 tumor cells preferentially colocalize with T-cells expressing CD49a, PD-1, and LAG3. **a** Schematic for topological analysis on the Visium spot hexagonal grid where the average expression of a gene is shown in a reference spot (gray) along with the average expression of the same gene in the spots located 1 spot away from the reference (red) or 2 spots away from the reference (orange) (top). Average expression of T-cell exhaustion and other immune markers surrounding spots enriched for each of 3 different Visium-derived epithelial signatures (bottom). * indicates *p* < 0.05 using a Fisher exact test for testing the association of expression of a given gene with enrichment of a given epithelial score. **b** Schematic of a MIBC tissue microarray (TMA) for multiplexed immunohistochemistry via CO-Detection by indEXing (CODEX). The CODEX panel consisted of 35 markers targeting epithelial, immune, and stromal cell types identified via snSeq analysis. **c** Median spatial distance per TMA spot of KRT13^+^ (yellow) or CDH12^+^ (blue) epithelial cells to the nearest B-cell, CD4^+^ T-cell, CD8^+^ T-cell, macrophage, or fibroblast. * - Mann-Whitney, two-sided, *p* < 0.05. *n* = 36, 63, 34, 63, 18, 40, 40, 66, 41, 68 for each box from left to right. Source data are available as a Source data file. **d** Voronoi diagrams of cellular neighborhoods (CN; top) and cell types (bottom). CN’s were identified by k-means clustering the distribution of cell types neighboring each cell. Spots were chosen based on the number of cells belonging to each of the 5 epithelial cell enriched CN’s. **e** Cellular diversity measured by the Shannon entropy of the cell types composing each of 5 epithelial enriched CN’s. * - Mann-Whitney, two-sided, *p* < 0.05. *n* = 42, 23, 63, 68, 67 for each box from left to right. Source data are available as a Source data file. **f** Marker intensity enrichment on CD8^+^ T-cells residing within each CN, compared against CD8^+^ T-cells residing in any other CN. Only Wilcoxon (two-sided) *p* < 0.05 are shown. **g** Sample images from *n* = 1 representative sample depicting a CD49a^+^ CD8^+^ T-cell (top), and PD-1^+^ CD8^+^ T-cell (bottom) in the immediate vicinity of CDH12^+^ epithelial cells in-situ. Scale bar - 11 μm. **h** Marker intensity enrichment on CDH12^+^ epithelial cells within each CDH12 enriched CN compared with CDH12^-^ epithelial cells within CN13 (left) or CDH12^+^ cells residing in any other CN (right). Only Wilcoxon (two-sided) *p* < 0.05 are shown. Boxplots are drawn as the inter-quartile range (IQR) with a line indicating the median, and outliers defined as points that fall outside of the range demarcated by 1.5*IQR.

To test the hypothesis that CDH12 epithelial cells attract T-cells, we first used the Visium spatial transcriptomics technology to investigate gene expression localization in tumors from our snSeq cohort. Visium-derived gene signatures closely matched with snSeq expression profiles, and distinct stromal and immune niches were also evident (Supplementary Fig. 6c, d). Topographic analysis found that areas enriched for a CDH12 signature were also enriched for CD8T with key markers of exhaustion (e.g. *PDCD1*, *LAG3*, *HAVCR2*) as well as the previously mentioned *ITGA1* (Fig. 5a). In contrast, spots enriched for a KRT13/UPK signature exhibited no T-cell gene enrichment.

To validate that CDH12 epithelial cells co-localize with T-cells at the single-cell level, we designed and executed a 35-plex IHC panel using the Co-detection by indexing (CODEX) platform on tumor tissue microarrays of the same tumor cohort (Fig. 5b and Supplementary Table 2)^40,41^. The tissue areas used in the microarray were specifically selected to harbor both tumor and stroma to allow the study of co-localization of tumor and non-tumor cells. We profiled a total of 75 cores across our patient cohort with ~360,000 epithelial cells, ~140,000 immune cells, and ~90,000 stromal cells passing quality control filtering. We successfully identified all of the major cellular populations including CDH12 epithelial and KRT13 epithelial cells based on expression of CDH12, CDH18, KRT13, and KRT17 (Supplementary Figs. 7, 8, and 9). We observed that the CDH12 population was significantly depleted for KRT13 expression while the KRT13 population was significantly depleted for CDH18 expression, suggesting KRT13 and CDH12 have different co-expression patterns at the protein level (Supplementary Figs. 7c and 10a).

### CDH12 cells define cellular niches with exhausted CD8 T-cells

Consistent with our Visium spatial transcriptomics results, we again observed closer proximity of CD8T to CDH12 epithelial cells than KRT13 epithelial cells using a k-nearest neighbor approach (Fig. 5c). More broadly, CDH12 epithelial cells resided in closer proximity to multiple immune cell types as well as fibroblasts. This suggested distinct spatial distributions for these two different populations. To formally address this, we utilized a cellular niche detection algorithm to identify Cellular Niches (CNs) in an unsupervised fashion^41^. CNs represent combinations of cell types that frequently co-localize across multiple tumors. Overall, we identified 20 total CNs comprising immune-enriched niches, some of which resembled tertiary lymphoid structures (TLS), stromal-enriched, and epithelial-enriched CNs (Supplementary Fig. 10b, c and Supplementary Fig. 11). Within the epithelial-enriched CNs, we identified 3 CNs that were significantly enriched for CDH12 epithelial cells, 2 of which were also enriched for CD8T. In contrast, we identified 2 CNs where the KRT13 epithelial cells were enriched, and they showed no enrichment for CD8T (Fig. 5d and Supplementary Fig. 10c). Additionally, the CDH12-enriched CNs were more diverse in terms of their constituent cell types than KRT13-enriched CNs, as assessed by Shannon entropy, a metric for diversity (Fig. 5e). This supported our original observations in that the CDH12 population resided in multiple spatially distinct niches that were immune-infiltrated, whereas the KRT13 population was restricted to niches resembling an immune “desert” phenotype.

We then asked how the identified CNs predict T-cell and epithelial cell phenotypes within them. CD8T residing within CDH12-enriched CNs expressed higher levels of CD49a (coded by ITGA1) (CN16), PD-1 (CN11 and CN14), and LAG3 (CN14) than CD8T residing in non-CDH12-enriched CNs (Fig. 5f, g). CDH12 cells within all 3 associated CNs had higher PD-L1 expression compared to epithelial cells in CN13, the most KRT13-enriched CN. In contrast, they expressed lower levels of PD-L2 (Fig. 5h, left). Interestingly, the CDH12 cells also expressed lower levels of Ki-67 compared to CN13, consistent with our snSeq findings and their potentially chemo-resistant nature. Among the 3 associated CNs, CN14 contained CDH12 cells with the highest PD-L1 and PD-L2 expression and this was consistent with CD8T in this niche having the highest expression of LAG3, which promotes a tolerogenic state in CD8T and exhaustion with PD-1 (Fig. 5f, h, right)^42,43^. Together, these data support the hypothesis that CDH12 epithelial cells reside near CD8T in part through CD49a interactions, and may promote T-cell exhaustion through PD-L1 and PD-L2^37–39^. This would partly explain the better response and survival for patients with high CDH12 signature scores when treated with atezolizumab.

## Discussion

**Fig. 6:**
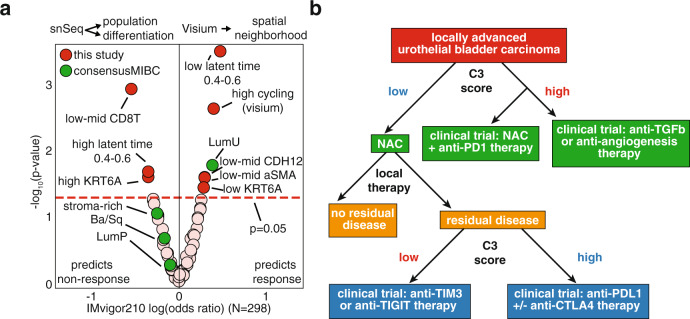
Gene signatures derived from single-nuclei sequencing and spatial transcriptomics outperforms bulk-RNA sequencing-based consensus classifiers in predicting response to immune checkpoint therapy. **a** Association of snSeq/Visium-derived signature scores, or consensus MIBC subtypes, with RECIST v1.1 response in IMvigor 210 Cohort 2 (*N* = 298, Fisher exact test). **b** Flow chart for incorporating a CDH12 score into clinical decision making for treatment-naïve and chemoresistant tumors.

In conclusion, we performed the first comprehensive profiling of MIBC at the single-nucleus level, which allowed us to elucidate the constituents of current molecular subtypes and to derive more therapeutically relevant molecular signatures with higher resolution. We identified both well-known epithelial phenotypes as well as a novel CDH12 phenotype that represents a previously undescribed poorly differentiated cellular state. This CDH12 “high” phenotype accurately predicts poor prognosis for patients treated with surgery as well as platinum-based neoadjuvant chemotherapy. It also successfully predicts better prognosis and higher response rates to PD-L1 blockade. We linked the chemoresistance of these cells to a reduced proliferative state, a highly fibrotic and vascularized tumor ecosystem, and expression of the chemoresistance gene *ALDH1A1*. However, these cells also express high levels of ligands for CD49a as well as PD-L1 and PD-L2, which combine to promote a microenvironment enriched for exhausted T-cells that likely become unleashed and benefit from immune checkpoint blockade. Through an extensive CODEX analysis, we confirmed the spatial proximity of CDH12 cells to CD49a-expressing, exhausted CD8T within unique cellular niches. Altogether, we derived gene signatures pertaining to specific cell populations, uroepithelial differentiation, and intra-tumoral spatial neighborhoods that provide superior therapeutic relevance than previous bulk-based subtypes (Fig. 6a). This subpopulation is remarkable for the degree it communicates with other cellular types and by virtue of this communication to establish distinct intratumor neighborhoods. Therefore, we propose to call this the Cell-Cell Communication (C3) subpopulation going forward and use its gene signature score (C3 score) in further studies.

Through these findings we speculate that gene expression profiling can serve to triage patients who would benefit from NAC (low C3 score) (Fig. 6b). Furthermore, these data indicate that anti-TGFβ/anti-angiogenesis strategies could be beneficial in high C3 score tumors. Residual tumors following NAC with low C3 score might benefit from targeting alternative immune checkpoint pathways such as TIM3 or TIGIT while those with high expression might benefit from single agent or combination ICT (Fig. 6b). This study paves the way for future analyses of the molecular mechanisms C3 cells employ to gain such unique predictive characteristics, and potentially for the development of inhibitors to enhance chemotherapy efficacy for tumors with high C3 scores. It also provides compelling rationale for a number of possible clinical trials based on tumors with high C3 scores prior to NAC as well as in patients with residual disease following NAC (Fig. 6b). While the IMvigor 210 trial results indicate that high C3 scores post-NAC predicts superior response to atezolizumab, paired pre- and post-NAC samples were not available. Therefore, we could not determine which of the post-NAC patients possessed low C3 scores prior to NAC and then determine if these had a different outcome compared to those patients with high C3 scores both before and after NAC. Thus, a prospective analysis which profiles how the evolutionary history of the tumor in response to NAC impacts response to atezolizumab would be insightful. We postulate that those tumors which start with a low C3 score and respond to NAC with increases in C3 scores would experience the most benefit from atezolizumab, as we showed that this is accompanied by an immune activation that might be prolonged with atezolizumab. Clinical assay development is also needed to address the practical application of a “low” versus “high” C3 score, which would entail establishing absolute standard curves for RNA/protein levels as RNA sequencing does not provide absolute quantitation. The addition of an IHC-based assay for enumerating C3/CD8T cellular niches similar to the ones we defined with CODEX may also prove useful to investigate the value of our findings in patient stratification for either NAC or checkpoint inhibitor therapy.

## Methods

### Research ethics

Urothelial tissue from twenty-five patients with high-grade muscle invasive bladder cancer (MIBC) and 4 patients without bladder cancer were obtained from patients who underwent surgery. All patients provided written informed consent, and no one receive neoadjuvant chemotherapy. All samples were immediately snap-frozen in liquid nitrogen and stored at −80 °C until used. The Research Ethics Committee of Cedars-Sinai Medical Center approved the study (Study00000542).

### Tumor and normal sample preparation

Nuclei were isolated from fresh frozen MIBC tumors using a method modified from a recent single-nuclei RNA-sequencing (snSeq) study^44^. The ST-SB buffer from that study was modified by removing Tween-20 and supplementing with 0.04 U/μL Protector RNase Inhibitor (Roche). Unless otherwise specified, all sample manipulation was performed on wet ice with wide-bore pipet tips (Rainin) and all centrifugations were performed with a swinging bucket rotor maintained at 4 °C for 5 min at 850 × *g*. In brief, the frozen tissue was transferred onto a plate on dry ice and crushed into ≤1 mm^3^ pieces. This was then transferred to a 2 mL dounce homogenizer (Kimble, cat: 885300-0002) on wet ice containing 1 mL of Nuclei EZ lysis buffer (Sigma, cat: NUC101). The tissue was then dounced approximately ×20 with Pestle A followed by ×20 with Pestle B. The lysis was then quenched by adding 1 mL of ST-SB. The sample was filtered through a pre-wetted 30 μm filter (Miltenyi Biotec, cat: 130-041-407) into a 15 mL conical tube. The homogenizer was rinsed 3× with 1 mL of ST-SB and this was transferred through the same 30 μm filter into the 15 mL conical tube. The sample was then centrifuged, the resulting supernatant removed, and the pellet resuspended with 500 μL of ST-SB. The sample was then passed through a pre-wetted 20μm filter (Miltenyi Biotec, cat: 130-101-812) into a 1.5 mL protein lo-bind microcentrifuge tube (Eppendorf, cat: 022431081) and centrifuged. At this point, Totalseq hashing antibodies (TotalSeq-A 0451 – 0456 anti-Nuclear Hashtag 1-6, Biolegend, clone Mab414, custom formulation provided by Biolegend) were also centrifuged at 14,000 × *g* for 10 min at 4 °C. The sample pellet was then resuspended in 100 μL of ST-SB and 10 μL of Human TruStain FcX block (Biolegend, cat: 422301) was added. The sample was pipet mixed and incubated at 4 °C for 5 min. Then 1.5 μg of the appropriate hashing antibody was added to the appropriate samples, pipet mixed, and incubated at 4 °C for 15 min. The samples were pipet mixed once halfway through this incubation. The samples were then washed 2× with 1 mL of ST-SB, pooled appropriately, and filtered through another 30 μm and 20 μm filter. Nuclei concentration was quantified by mixing an aliquot of the sample with DAPI at a final concentration of 0.025 mg/mL in H_2_O. Samples were finally processed according to 10× Genomics protocol for the 3’ v3.1 assay and were super-loaded to target of 20,000 nuclei recovery. We observed that nuclei yield less total cDNA than cells, therefore we increased the first cDNA amplification cycle number by 2. Hashing libraries were generated according to the Biolegend Totalseq protocol for the 3’ v3.1 assay. In total, 57 samples from 25 patients were processed.

Nuclei were isolated from histologically normal bladder tissue using the same protocol as above, but without hashing antibodies. Therefore, each sample was run in its own 10x Genomics reaction. In total, 4 samples from 3 patients were processed, with 3 samples originating from patients with urothelial carcinoma or leiomyosarcoma (taken distant from the involved site and verified by a trained pathologist to be uninvolved), and 1 sample originating from a healthy bladder. All samples were sequenced by the Cedars-Sinai Applied Genomics, Computation & Translational Core on a Novaseq to a sequencing saturation of approximately 60%. Samples were processed with CellRanger (10X genomics, v3.0.2) using a pre-mrna reference based on the GRCh38-3.0.0 reference in a manner similar to a recent snSeq study^45^. Hashing libraries were aligned using the Cite-seq-count program (v1.4.3) with the cell barcodes from the CellRanger output as the barcode whitelist. The UMI counts from Cite-seq-count were then used for demultiplexing the MIBC samples using a combination of the Seurat HTOdemux function and a secondary custom script in MATLAB. The secondary script was used to recover nuclei that were identified as negative for all hashtags by the HTOdemux function, but actually passed the minimum number of counts identified by the HTOdemux function for one and only one hashtag. All nuclei that were determined to be doublets or that remained negative after the recovery step were then removed from subsequent analyses. Since the histologically normal samples were not hashed, putative doublet nuclei were identified using Scrublet (v0.2.1)^46^ from the filtered feature barcode matrices produced by CellRanger. Scrublet was run using the 10% highest variable genes, identified using the Scanpy (scanpy.pp.highly_variable_genes function; scanpy v1.5.1)^47^, with an expected doublet rate of 10%. Nuclei were scored as candidate doublets by Scrublet and removed if their doublet score exceeded 0.25. Finally, for all samples, nuclei with more than 10% of their UMIs mapped to mitochondrial genes were removed, and the top and bottom 5% of nuclei based on number of unique genes and number of UMI were removed.

### Visium sample preparation

Tissue optimization was performed on one representative MIBC sample from the cohort used in this study, and the optimal permeabilization time was determined to be 24 minutes. Then 4 samples were cryosectioned at 10μm and processed according to the 10x Visium protocol. Samples were sequenced by Illumina to a sequencing saturation of approximately 90%. Samples were processed with SpaceRanger (10X genomics, v1.1.0) using the same pre-mrna reference as for the snSeq data analysis to improve consistency between the two datasets. Visium spots were filtered to have at least 1,250 total UMI and less than 10% of their UMIs mapped to mitochondrial genes. Genes that were not detected in at least 4 spots were removed.

### Public bulk RNA-seq datasets: TCGA, IMvigor 210, neoadjuvant chemotherapy (NAC)

Bladder urothelial carcinoma Illumina Hi-Seq counts from The Cancer Genome Atlas (TCGA) were downloaded from the Genomic Data Commons (GDC) data portal, and corresponding clinical annotation including survival information was accessed via the TCGA Clinical Data Resource^48^. Consensus MIBC classifications of TCGA cases were obtained from the consensus MIBC study^27^. Only untreated high-grade muscle invasive cases with outcomes were analyzed (*N* = 259). RNA-seq and sample annotations including overall survival from the IMvigor 210 trial were accessed as described^49^. For survival analysis of IMvigor 210, only samples from Cohort 2 which were annotated as originating from bladder in the pre-chemotherapy (*N* = 100) or the post-chemotherapy (*N* = 53) setting were used. For pathological response analysis of IMvigor 210, only samples from Cohort 2 which had pathological response information and were annotated as originating from bladder in the post-chemotherapy setting (*N* = 51) were used. For the comparison of response prediction shown in Fig. 5c, all samples from Cohort 2 of IMvigor 210 with pathological response information (*N* = 298) were used to facilitate comparison with the consensus MIBC results which were previously published using those samples. After Illumina Hi-Seq counts were obtained from the respective repositories, the raw counts were counts-per-million normalized and log-transformed. Affymetrix array data corresponding to a trial of neoadjuvant cisplatin-based chemotherapy in MIBC was downloaded from GEO (GSE124305 and GSE87304). Array data were normalized using the RMA method from the oligo R package (v1.52.1).

### Single cell dimensionality reduction, clustering, and subtyping

Dimensionality reduction and cell type assignment were carried out in a two-step process. Tumor and normal cohorts were clustered and subtyped separately. First, all cohort cells were used to fit a single cell Variational Inference model (scVI v0.6.8)^50^, resulting in a 128-dimensional representation of cell phenotypes. The scVI latent space was further projected into a 2-dimensional space for visualization by Uniform Manifold Approximation and Projection (UMAP, Rapids.ai cuml v0.12.0)^51,52^. Unsupervised clustering was performed on the scVI latent space via the leiden community detection algorithm (cugraph v0.17, resolution = 0.6) and clusters were labelled as broadly epithelial, fibroblast, immune, or endothelial using a panel of marker genes gleaned from the literature. Clusters that could not be clearly annotated as a specific cell type or clusters that expressed combinations of lineage-defining markers that are not known to be co-expressed were removed from further analysis. Each broad cell type was then sub-clustered by again applying scVI and the Leiden algorithm. To identify marker genes for detailed subtyping, differential gene expression analysis was applied between sub-clusters in a 1-vs-all fashion (scanpy, Wilcoxon method). Cell types were assigned based on alignment of top differentially expressed genes with marker gene sets gathered from the literature. Gene set scores from published MIBC subtyping and tumor stem cell studies were evaluated for each epithelial cell by comparing the average expression to that of similar-expression genes^53^.

To derive gene sets specific to each cell subtype identified in snSeq we applied differential expression analysis separately within the 3 broad cell compartments (epithelial, fibroblast, and immune). For each compartment, a differential expression test was performed genome-wide for each specific subtype against all others subtypes in that compartment (e.g. KRT epithelial vs CDH12 epithelial, cycling epithelial, etc.). The top 200 up-regulated genes for each subtype according to the scanpy “rank_genes_groups” tool’s “score” column were taken as putative markers for that subtype. To break ties in cases when one gene was assigned as a marker to multiple subtypes, the gene was ultimately assigned to the subtype with the higher “score”. These gene signatures can be found in Supplementary Data File 3.

### SCENIC regulon analysis and gene co-expression modules

To interrogate active transcriptional networks within each epithelial cell subtype we performed gene co-expression module analysis and single-cell regulatory network inference and clustering (pySCENIC v0.10.0)^15^. SCENIC analysis was performed with 6,979 highly variable genes using a curated list of human transcription factors, and cisTarget database scoring motif enrichment up to 10 kilobases up and downstream of transcription start sites. To complete the SCENIC workflow, AUCell scores were calculated for each identified regulon.

Gene co-expression modules for tumor epithelial nuclei were derived from the genome-wide pairwise gene Pearson correlations calculated from library size-normalized, log-transformed counts. Genes were filtered first based on being differentially expressed across clusters (FDR ≤ 0.05 and absolute log fold change ≥ 0.3) and then based on a minimum number (*N* = 5) of correlations above an absolute correlation threshold (Corr = 0.4). Genes were clustered according to Pearson correlation and modules were partitioned by hierarchical clustering (scipy, metric = Euclidean). Module genes were queried for Gene Ontology (GO) term enrichment using gprofiler via scanpy^54^. For visualization, individual genes were associated to the epithelial cell subtype with maximum expression of that gene.

### RNA velocity and tumor nearest normal neighbor identification

Alignment for RNA velocity analysis was performed using the velocyto package^23^, and downstream velocity analysis was performed using scVelo (v0.17.15)^22^. The same genome annotation files used for CellRanger were used for alignment, and the GRCh38 repeat mask files were downloaded from the UCSC genome browser. Cells that had previously passed QC and were subtyped in the previous gene expression analyses were extracted from the velocyto output.

Normal epithelial nuclei were analyzed individually with scVelo. Gene expression moments were calculated on the top 5,000 highly variable genes with at least 20 combined counts using the UMAP method. RNA velocity was run using scVelo’s dynamical model. Next, we sought to find the cell from the normal samples that was nearest to each tumor epithelial cell in gene expression space. The top 500 genes correlating gene expression with the latent time (minimum correlation 0.3) were identified from each normal sample and aggregated (total 1,118 unique genes). Using the library size-normalized, log-transformed counts of these latent time genes we proceeded by comparing each tumor epithelial cell with each normal epithelial cell by calculating the L1 norm of the difference of normalized gene expression. Each tumor epithelial cell inherited the latent time of its nearest neighbor normal cell defined as the normal cell with the minimum L1 norm. Latent time gene signatures were derived by first binning tumor epithelial cells into 5 evenly spaced time intervals according to their predicted latent time. Differential expression was performed to recover the top 200 differentially expressed genes for cells within each time interval versus all other time intervals in a 1-vs-all fashion (scanpy, Wilcoxon method). In the event that a gene appeared in the top 200 for more than one-time interval, the gene was assigned to the signature of the interval with the highest differential expression score.

### Ligand-Receptor interaction analysis

Receptor activity scores were based on expression of signaling proteins and gene regulation targets downstream of receptor activation^29^. A curated table of ligand-receptor pairs was obtained from SingleCellSignalR^30^. We first assembled gene signatures describing receptor activity by collecting protein-protein signaling connections and gene regulatory associations included in the NicheNet graphs. Ultimately, 75 receptors that failed to accumulate signatures of at least 5 genes were excluded from further analysis, leaving a total of 675 receptors, and 2,886 total ligand-receptor pairs to be interrogated. The receptor activity was defined as the average absolute deviation of receptor signature genes from the average expression of those genes in a background composed of the same broad cell type (epithelial, fibroblast, lymphoid, myeloid).

Ligand-receptor interactions were determined based on the expression of the ligand in a sender population of cells and the concurrent activation of the corresponding receptor in a receiving population of cells. To perform a general interaction analysis, we first pooled cells by subtype across all tumor samples. To determine available ligands that were enriched in individual subtypes, we performed differential expression analysis (scanpy, Wilcoxon method) of ligand genes for each subtype against cells within the same broad cell type. Available ligands for a sending population were those that met a minimum log fold change of 0.5 and maximum adjusted *p*-value of 0.05. Similarly, receptor activities were tested for enrichment in each subtype relative to a background of the same broad cell type. Active receptors were called according to a minimum log fold change of 0.25 and maximum adjusted *p*-value of 0.05. All ligands and receptors were required to be expressed in at least 10% of sending or receiving cells respectively. Candidate ligand-receptor pairs were assessed from the available ligands and active receptor sets. Finally, candidate ligand-receptor pairs were subjected to a spatial co-expression filter. Spatially co-expressed ligand-receptor pairs were determined in the spatial transcriptomics dataset. A ligand-receptor pair was called spatially co-expressed if, within at least 1 tumor, 25% of “spots” exhibiting the ligand expression (UMI > 0) also had receptor expression (UMI > 0). Ligand-receptor pairs were visualized with Circos plots. Each plot included heatmap tracks of standardized ligand expression in one sending subtype and standardized receptor activity in several receiving subtypes. Interaction potential was defined as the product of average ligand expression with average receptor score and visualized as links connecting ligand to receptor. Ribbon transparency was determined by the scaled interaction potential according to transparency = min(0.9, 1−(potential/potentialmax)^2^) so that the highest potential interaction was the least transparent and a maximum transparency of 90% was imposed to ensure all ribbons were visible.

### ssGSEA, Kaplan-Meier analysis, and differential gene expression for bulk RNA-seq

Gene set enrichment of the tumor single-cell subtype signatures and latent time signatures was assessed in each of the bulk RNA-seq samples from the TCGA and IMvigor 210 cohorts, and in the Affymetrix array data of the Black cohort. TCGA and IMvigor 210 samples were scored by single sample Gene Set Enrichment Analysis (ssGSEA, package GSEApy v0.10.1)^55^. The neoadjuvant chemotherapy cases were scored with Gene Set Variation Analysis (package GSVA v1.36.2)^56^. Samples within each cohort were grouped by score quartiles and Kaplan-Meier survival plots were fit using the right-censored overall survival or disease-free survival times (lifelines version 0.25.4)^57^. Significance was assessed between the survival curves of the first and fourth quartiles using a log-rank test. Differential gene expression analysis for the neoadjuvant chemotherapy dataset was performed using the limma R package (v3.44.3).

### Spatial gene signatures and association with T-cell exhaustion markers

Gene co-expression modules for the Visium spots were obtained in a similar fashion as for the snSeq epithelial analysis, however in this case differential gene expression analysis was performed on each sample using the SpatialDE package (v1.1.3)^58^ and genes with FDR ≤ 0.05 were combined across samples. Then the same cutoffs from the snSeq analysis were applied except the fold change cutoff was removed. The resulting gene co-expression modules were then annotated based on their relation to the snSeq dataset, e.g. the module whose gene signature was enriched in the CDH12 nuclei was labeled as CDH12-enriched.

Visium field expression profiles (Fig. 4G) were generated by taking the top 5th percentile of spots for a given module as the reference spots, and then averaging the expression of spots in rings around the reference spot. The coordinates for the ring are as follows: (x-(k+1)),(y+(k+1)); (x-(k+1)),(y-(k+1)); (x),(y+(k+2)); (x),(y-(k+2)); (x+(k+1)),(y+(k+1)); (x+(k+1)),(y-(k+1)); where (x,y) are the coordinates for the reference spot and k is the number of spots away from the reference. The figure shows the average of these profiles across all of the reference spots considered and standardized across the modules.

Visium spots were tested for concurrent enrichment of expression profile scores and gene expression by contrasting spots in the top 5^th^ and bottom 5^th^ percentile of module scores. A contingency table was constructed by counting the number of spots with gene expression in the top 5th and bottom 95th percentile and Fisher’s exact test (scipy v1.4.1, fisher_exact, one-sided) was performed on the contingency table.

### Immunohistochemistry

Immunohistochemistry was performed on sections taken from FFPE blocks that were made from adjacent pieces of the same tumors from the snSeq cohort. Briefly, sections were deparaffinized and rehydrated, antigen retrieval was performed using a pressure cooker and 1x Universal HIER buffer (Abcam, cat: ab208572), then blocked in protein blocking buffer (Abcam, cat: ab64226) for 1 h at room temperature. Sections were then washed and incubated with primary antibodies at 4 °C overnight. The primary antibodies used were as follows (all dilutions were performed with protein blocking buffer): KRT13 (Abcam, cat: ab239918, clone EPR3671, 1:100), KRT17 (Abcam, cat: ab212553, clone KRT17/778, 1:100), CDH12 (LSBio, cat: LS-B11408-100, rabbit polyclonal, 1:100), and CDH18 (Thermo-Fisher Scientific, cat: H00001016-M01, clone 6F7, 1:50). Sections were then washed and incubated with the appropriate fluorophore-conjugated secondary antibodies at room temperature for 1 hour. Secondary antibodies used were as follows (all dilutions were performed with protein blocking buffer): Donkey anti-mouse IgG AF568 (Thermo Fisher Scientific, cat: A10037, 1:500) and goat anti-rabbit IgG AF488 (Thermo Fisher Scientific, cat: A11008, 1:500). Sections were finally washed, mounted with Vectashield containing DAPI (Vector Laboratories, cat: H-1200), and imaged using a Leica DMi8 equipped with a Lumencor SOLA SE U-nIR LED and Hamamatsu Orca Flash 4.0 v3.

### Co-detection by indexing (CODEX) of MIBC tumor microarrays

Tumor microarrays (TMAs) were prepared from 1 mm punches taken from FFPE blocks that were made from adjacent pieces of the same tumors from the snSeq cohort. If possible, 3 punches were taken from each tumor with 1 punch per tumor, per TMA, resulting in 3 final TMAs. Punches were taken from areas of the tumor that were annotated on H&E to contain both tumor and stroma as annotated by a trained pathologist. Sections from each of these 3 TMAs were then collected onto poly-L-lysine-coated coverslips, which were prepared according to the Akoya Biosciences CODEX protocol^40^. Sections were then deparaffinized and rehydrated, and antigen retrieval was performed in a similar manner to the IHC protocol. Sections were then quenched for autofluorescence using a protocol adapted from Du et al.^59^. Subsequently, sections were stained and imaged according to the Akoya Biosciences CODEX protocol. Details regarding primary antibodies and imaging conditions can be found in Supplementary Table 2. Imaging was performed using a Leica DMi8 equipped with a 20x objective, Lumencor SOLA SE U-nIR LED, and Hamamatsu Orca Flash 4.0 v3.

Primary antibodies were initially screened by performing standard IHC, as above, on MIBC tumor sections to verify positive staining. Primary antibodies were then conjugated to their corresponding barcodes according to the Akoya Biosciences CODEX antibody conjugation protocol. Conjugated antibodies were then titrated by performing CODEX staining on a TMA section using the full panel diluted at either ×50, ×100, ×200, or ×400. The dilution that resulted in the optimal signal-to-noise ratio was determined for each antibody individually. The final dilutions obtained from this titration can be found in Supplementary Table 2.

### CODEX data pre-processing

Images were processed with custom software. To process raw CODEX images, 5 preprocessing operations were applied in this order: extended depth of field (EDOF), shading correction, cycle alignment, background subtraction and tile stitching, described briefly here.

1. An EDOF image was produced from the z-stack for each tile where each position is taken from the z-plane most in focus.
2. The CIDRE method^60^ of optical shading correction was applied to each channel of each imaging cycle.
3. An image registration transformation was estimated between the first cycle DAPI channel and the DAPI of each subsequent cycle. For each cycle, the registration parameters were saved and applied to all other channels from the same cycle.
4. Blank cycles were used to subtract background from each channel.
5. Finally, neighboring tiles were stitched by applying a registration between the overlapping areas between two tiles. First the two tiles with the best naive overlap were stitched by applying the appropriate registration shift to one of the tiles. Stitching then proceeded with the next two most nearly aligned tiles, until all tiles were merged. Since each cycle was previously aligned to the first cycle’s DAPI channel, the registrations used for tile stitching were estimated once on the first DAPI and reused for subsequent channels and cycles.

To obtain nuclear segmentations we applied a pre-trained StarDist model^61^ to the first cycle DAPI image. The model weights of the 2D 2018 Data Science Bowl model released by the original StarDist authors were fine-tuned using a training set of nuclei imaged on our CODEX platform. A “ring percentage” metric was also developed for relevant markers to differentiate cells expressing the marker from adjacent cells whose masks may contain a portion of the signal from the positive neighbor. For surface markers the assumption was, truly positive cells would display signals in a ring-like morphology, while neighboring cells with overlapping masks would not. To quantify cells exhibiting a ring-like pattern, we defined the “ring percentage” by examining the pixels in a ring around the nuclear segmentation contour, and tallying the percentage of these pixels that were positive for the markers CD45, CD3e, CD8, CD4, CD45RA, CD45RO, CDH12, KRT13, KRT17, CD20, ERBB2, and PanCytoK, defined as intensity greater than 20. Lastly, a whole-cell or “membrane” segmentation was obtained expanding the nuclear segmentation area by morphological dilation, without introducing overlaps in adjacent nuclei. The average intensities under each nuclear mask and membrane mask were extracted for each cell to be used for cell type assignment. A Hematoxylin and Eosin stained slide accompanying each of the 3 TMA’s was examined by a pathologist and spots identified as necrotic, or with extensive tearing or cautery artifacts were excluded from further analysis.

### CODEX cell type identification

A multi-step strategy was used to assign specific subtypes to single cells by first gating average marker intensity, then applying a k-Nearest Neighbor (kNN) classifier. First, the initial set of 615,171 segmented cells was filtered for low-quality cells indicating errant segmentations or non-specific staining artifacts with three separate gates: low DAPI intensity (filtered 2,501 cells), low total marker expression (filtered 17,597 cells), and high multiple marker expression (filtered 12,547 cells). Cells were manually gated based on intensity of PanCytoK, CD45, aSMA, CD31, CD20, CDH12, CDH18, CD68, CD3e, CD8, and CD4 into a training set consisting of the broad cell types: Epithelial, Epithelial KRT, Epithelial CDH, Stromal, Endothelial, general CD45+ immune, Bcell, CD8T, CD4T, and Macrophage. Further selection based on the “ring percentage” feature described above was applied to filter the gated populations using the applicable markers. For this initial classification, the special “blank” and “saturated” classes were retained. The cells that fell into these categories during this initial classification were dealt with in a later step. To account for imbalance in the training set collected, each category was uniformly subsampled to 2,500 training cells, unless fewer than 2,500 training cells were collected in which case all cells were used for that category. In all, a training set of 32,500 cells was used for initial cell typing. 50 features per cell were used for kNN classification: aSMA, CD45, PDGFRb, CD68, CD31, HLA-DR, UPK3, GATA3, CD3e, CDH18, CDH12, KRT13, KRT17, CK5-6, KRT20, CD20, CD8, CD4, and PanCytoK “membrane” and “nuclei” mean intensity features (38), and all “ring percentage” features (12). Features were scaled with the robust scaling method in scikit-learn to normalize the inter-quartile ranges of each feature. A kNN classifier (cuML, version 0.17) was trained on the whole training set using 200 neighbors and uniform weighting. Cells initially classified as CD8T or CD4T were next used in a second phase of T-cell specific gating to identify activated CD8T (CD45RA^hi^, CD69^hi^ /CD45RO^lo^, PD-1^lo^), terminally differentiated CD8T (PD-1^hi^/CD45RO^lo^, CD69^lo^), resident memory CD8T (CD49a^hi^, CD103^hi^ / FOXP3^lo^), and regulatory CD4T (FOXP3^hi^/CD49a^lo^, CD103^lo^). In keeping with the aforementioned class balancing procedure, up to 500 cells from each Tcell subset were randomly selected for training, and up to 500 CD8T and CD4T cells not included in the specific subtyping were also included. Thus, a total of 2,445 cells were used for training a second T-cell specific kNN classifier with 100 neighbors.

The final phase of subtype classification was to assign subtypes to those cells still labelled “blank”, “saturated”, or non-descript “Immune”. All cells with a final subtype were used as potential training cells for 10 rounds of classification. Each round, 500 of each subtype were randomly selected as training cells for a kNN classifier with 20 neighbors. The rescued cells were assigned the most frequently predicted subtype across the 10 rounds. Rescued cells assigned to non-immune subtypes were accepted, however, rescued immune cells were rejected and filtered from the dataset. Finally, Epithelial KRT13+ and KRT17+ cells were selected by manually gating KRT13 and KRT17 intensity from all classified Epithelial cells. Ultimately, 598,327 cells were assigned a celltype and subtype annotation and included for further analysis. Marker intensity was visualized using a dot plot where the hue of the dots represented the log fold change of that marker in a particular subtype versus all other cells, and the size of the dot represents a Wilcoxon test p-value (scipy, version 1.6.0).

### CODEX niche detection and spatial analysis

Niches were identified according to the subtype distribution of the k = 10 nearest cells, with a maximum distance of 200 in image coordinates. Each cell’s neighborhood profile was tallied as the percentage of each broad cell type (Epithelial, Epithelial CDH, Stromal, Endothelial, Macrophage, Bcell, CD8T and CD4T) within each cell’s 10 nearest neighbors by Euclidean distance, and including the reference cell’s celltype. A cellular niche (CN) represents groups of cells with similar neighborhood profiles. Using an iterative classifier-based approach we identified an optimal number of CN’s. A k-means clustering (cuML, version 0.17) was performed with several values of k. For each k value, all cell niches were clustered, then divided into 1/2 training and 1/2 hold out partitions, then a logistic regression classifier (cuML, version 0.17) was fit on each CN in a 1-versus-all fashion. The area under the receiver operating characteristic curve (AUC) for each of these classifiers was evaluated using the held out partition. The average AUC for each k was plotted. The value k = 20 was chosen as a value providing a reasonable number of niches with good individual predictability. The 1-vs-all logistic regression model coefficients were used to assign labels based on predictive cell types for each niche. Two niches with similar composition were merged, yielding 19 final CN’s for further analysis. Subsequently, the specific subtype membership within each CN was examined using a Fisher’s exact test.

The cellular niche diversity was defined as the Shannon entropy (Eq. 1) of the cells composing a CN, i.e. the cells assigned to the CN, and all of the cells included in computing those neighbor profiles. Only unique cells were considered. For a set of CN cells consisting of $n$ subtypes, $P\left({x}_{i}\right)$ represents the frequency of the $i$th subtype amongst the set, and the Shannon entropy is given by Eq. 1. A large value of Shannon entropy indicates diversity in the cell subtypes, whereas a low value indicates a lack of diversity, or that the CN is dominated by a few subtypes.

1 $$S=-\mathop{\sum }\limits_{i=1}^{n}P\left({x}_{i}\right){\log }P\left({x}_{i}\right)$$

Relative marker enrichment between CN’s was evaluated with a Wilcoxon test of marker intensity on a specific subtype of cells residing within a particular CN compared with intensity on a subtype of cells residing in another CN. Lastly, direct spatial proximity between two cell types was evaluated per spot as the median distance between each instance of a query cell type to the nearest instance of a target cell type. A Mann–Whitney test was used to assess a difference in these distances across all spots in all TMA’s. In all analyses, only spots with at least 25 examples of all cell types, subtypes, or CNs being examined were evaluated.

### Reporting summary

Further information on research design is available in the Nature Research Reporting Summary linked to this article.

## Supplementary information

Supplementary Information

Description of Additional Supplementary File

Supplementary Data 1

Supplementary Data 2

Supplementary Data 3

Reporting Summary

## Data Availability

The following datasets were generated in this study. Single-nuclei RNA-seq and HTO data have been deposited in the GEO database under accession code GSE169379. Visium data have been deposited in the GEO database under accession code GSE171351. CODEX processed data are available through figshare from the following links: https://figshare.com/s/4610a15363c8306dfa36, https://figshare.com/s/2005255a8b65de23109f, https://figshare.com/s/1d8c7ed76d4b3222ada4. CODEX raw data are available from the corresponding authors upon reasonable request. The following datasets are publicly available. Bladder urothelial carcinoma Illumina Hi-Seq counts from The Cancer Genome Atlas (TCGA) were downloaded from the Genomic Data Commons (GDC) data portal, and corresponding clinical annotation including survival information was accessed via the TCGA Clinical Data Resource. Data from the IMvigor210 trial were obtained from the IMvigor210CoreBiologies R package, made freely available by the authors of the trial manuscript. Affymetrix array data corresponding to a trial of neoadjuvant cisplatin-based chemotherapy in MIBC was downloaded from GEO (GSE124305 and GSE87304). The remaining data are available within the Article, Supplementary Information, or Source Data file. Source data are provided with this paper.

## Source data

Source Data

## Code availability

Software packages, notebooks, and scripts used for analysis are available at https://github.com/KnottLab/bladder-snSeq. Custom MATLAB code for CODEX preprocessing is available at https://github.com/KnottLab/codex. The corresponding DOIs are as follows, analysis scripts: 10.5281/zenodo.5115212 and CODEX preprocessing: 10.5281/zenodo.5115210.
