# Supplementary material for: An N-Cadherin 2 expressing epithelial cell subpopulation predicts response to surgery, chemotherapy and immunotherapy in bladder cancer
Source: Nat Commun. 2021 Aug 12;12:4906. doi: 10.1038/s41467-021-25103-7 (PMC8361097; doi:10.1038/s41467-021-25103-7)
Supplement: Supplementary file 1 — Supplementary Information [file 41467_2021_25103_MOESM1_ESM.pdf]

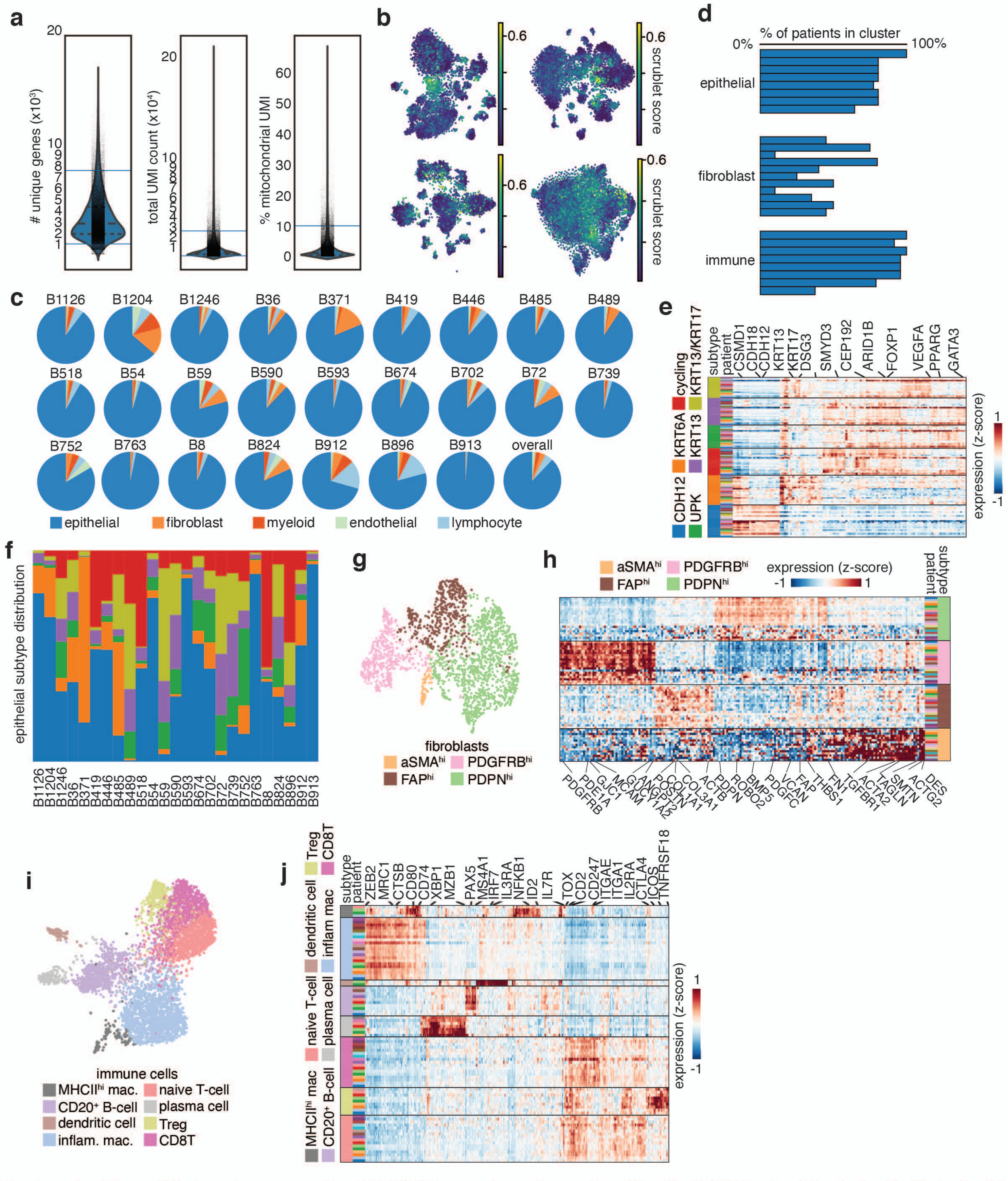

Supplementary Figure 1) Single nucleus sequencing of the MIBC tumor microenvironment. a, QC metrics for MIBC snSeq dataset where the blue horizontal lines represent the top and bottom 5th percentiles for the number of unique genes and total UMI or the 10% threshold for the UMI percent mitochondrial-coding genes. **b,** Scrublet scores for each of the histologically-normal bladder samples. **c**, snSeq population proportions in 25 muscle invasive bladder tumors, and the overall combined population proportions. **d**, Percent of patients analyzed that are represented in each of the unsupervised clusters using the scVI method. **e,** Average gene expression per patient of marker genes for each epithelial population in **Main Fig 1d**. **f**, Epithelial population distribution for each patient analyzed, colors correspond with **e**. **g,** UMAP of fibroblasts (2,075 nuclei) from MIBC tumors colored by unsupervised clustering**. h,** UMAP of immune cells (6,121 nuclei) from MIBC tumors colored by unsupervised clustering. **i**, Average gene expression per patient of marker genes for each fibroblast population in **g**. **j**, Average gene expression per patient of marker genes for each immune population in **h**. Gene expression values shown as log(CP10k + 1), heatmaps show average gene expression per cluster and z-scored within each patient.

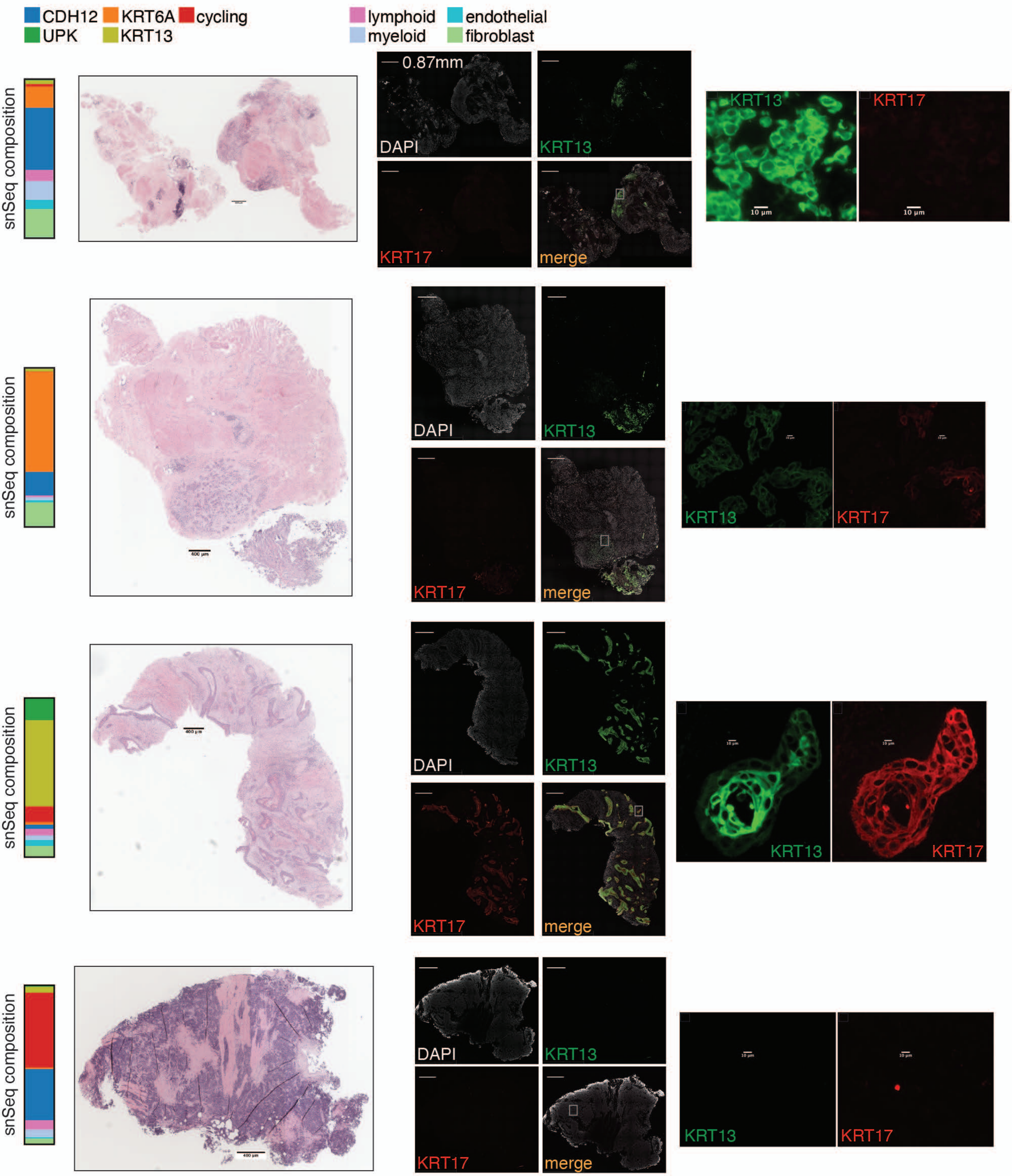

Supplementary Figure 2) Immunohistochemistry validation of KRT13 and KRT17 expression in 4 tumors from MIBC cohort. Scale bars are 400µm, 870µm, and 10µm in the left, middle, and right columns, respectively.

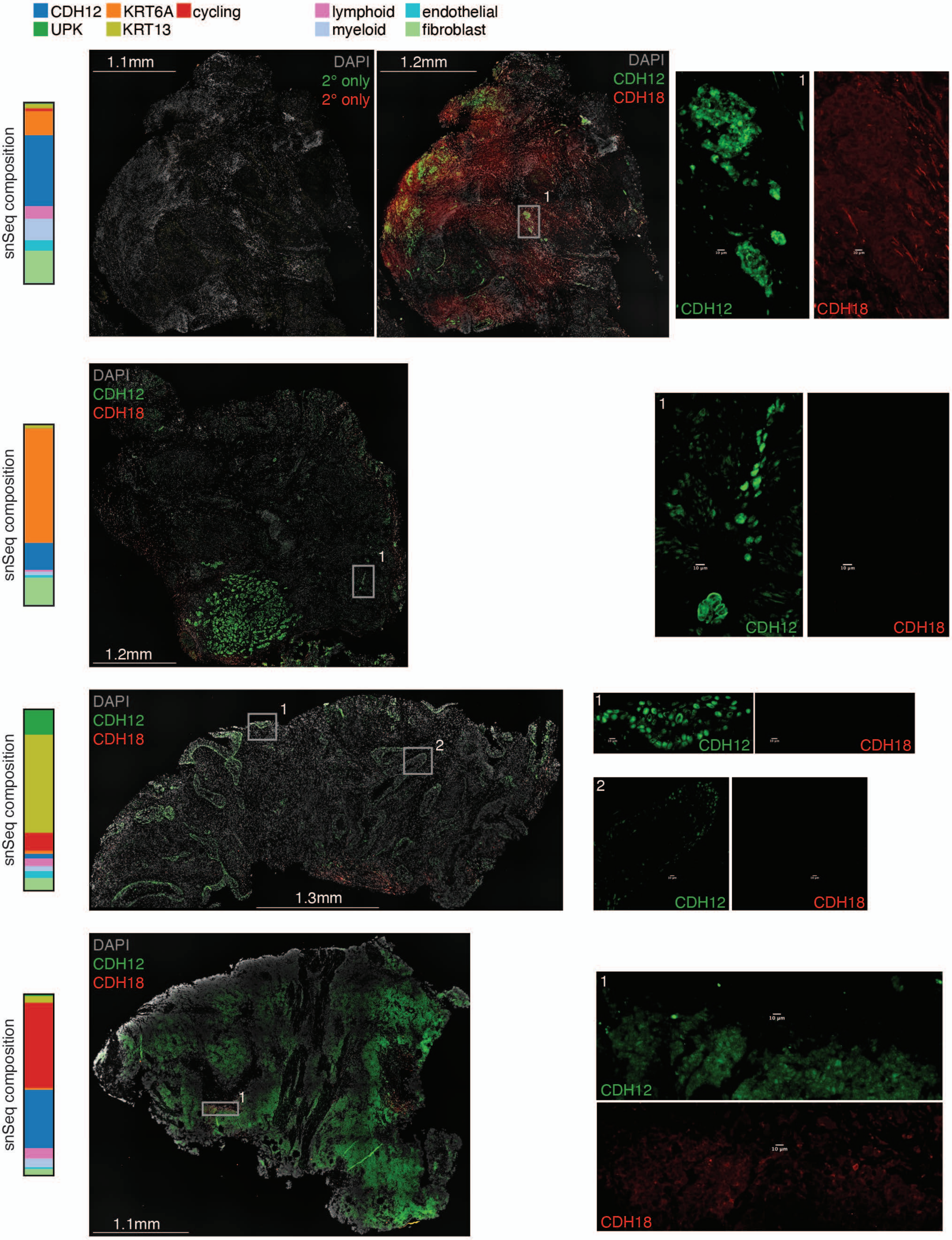

Supplementary Figure 3) Immunohistochemistry validation of CDH12 and CDH18 expression in 4 tumors from MIBC cohort. Scale bars in the left column are shown with their respective lengths and scale bars in the right column are 10μm.

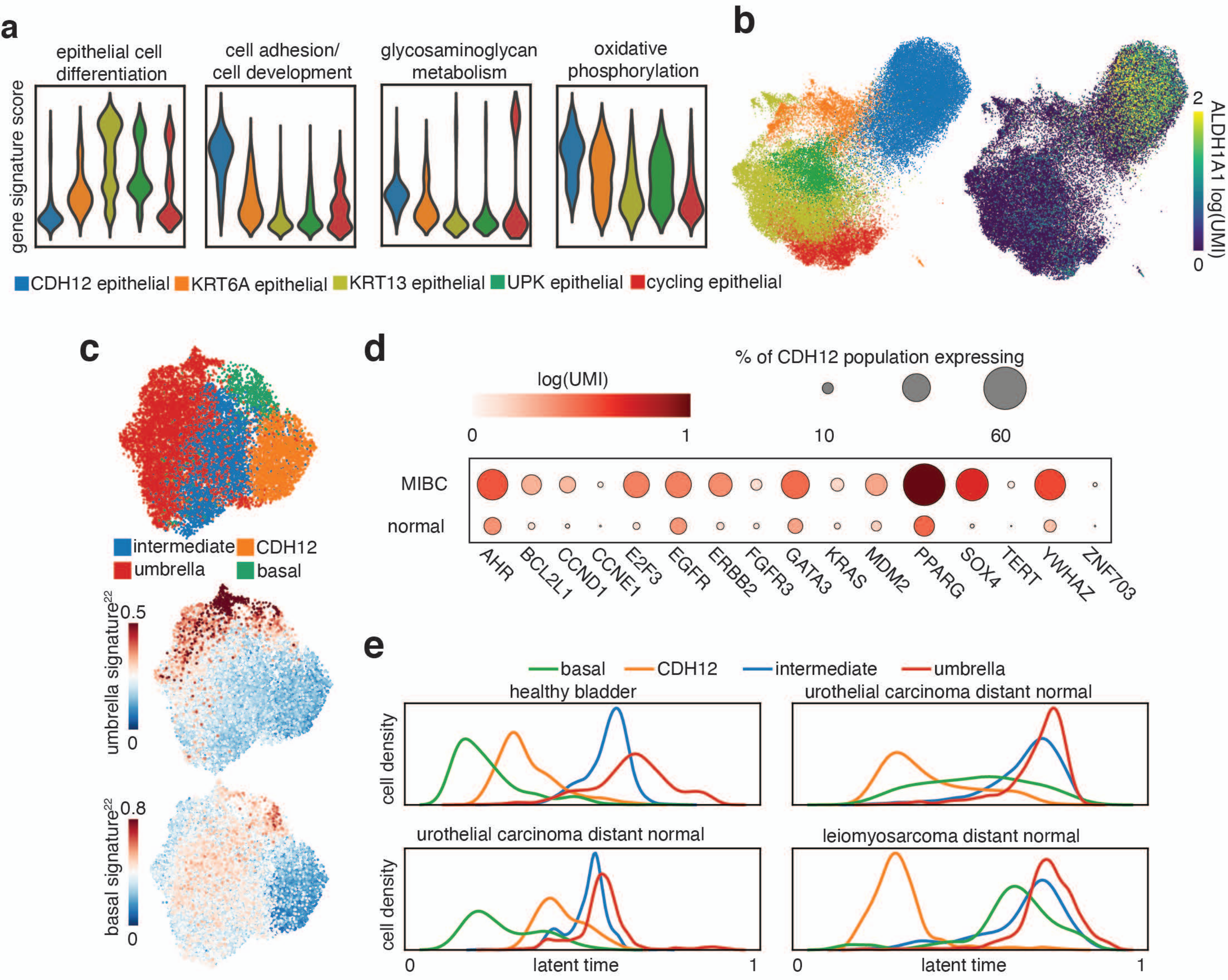

Supplementary Figure 4) Single nucleus sequencing of healthy bladders. a, Gene signature scores of co-expression modules identified in **Main Fig. 1g** separated by epithelial population. **b,** Epithelial populations (left, same as **Main Fig 1d**) and *ALDH1A1* expression in the MIBC epithelial nuclei (right). **c,** Normal bladder epithelial populations (left, same as **Main Fig. 2a**) and umbrella (middle) and basal (right) cell gene signature scores in 12,819 epithelial nuclei from histologically-normal bladders. **d**, Expression of genes commonly overexpressed in bladder cancers in MIBC versus normal bladder CDH12 populations. **e**, Density plots of the healthy bladder epithelial populations ordered by latent time in each of the 4 histologically-normal bladder tissues that were profiled.

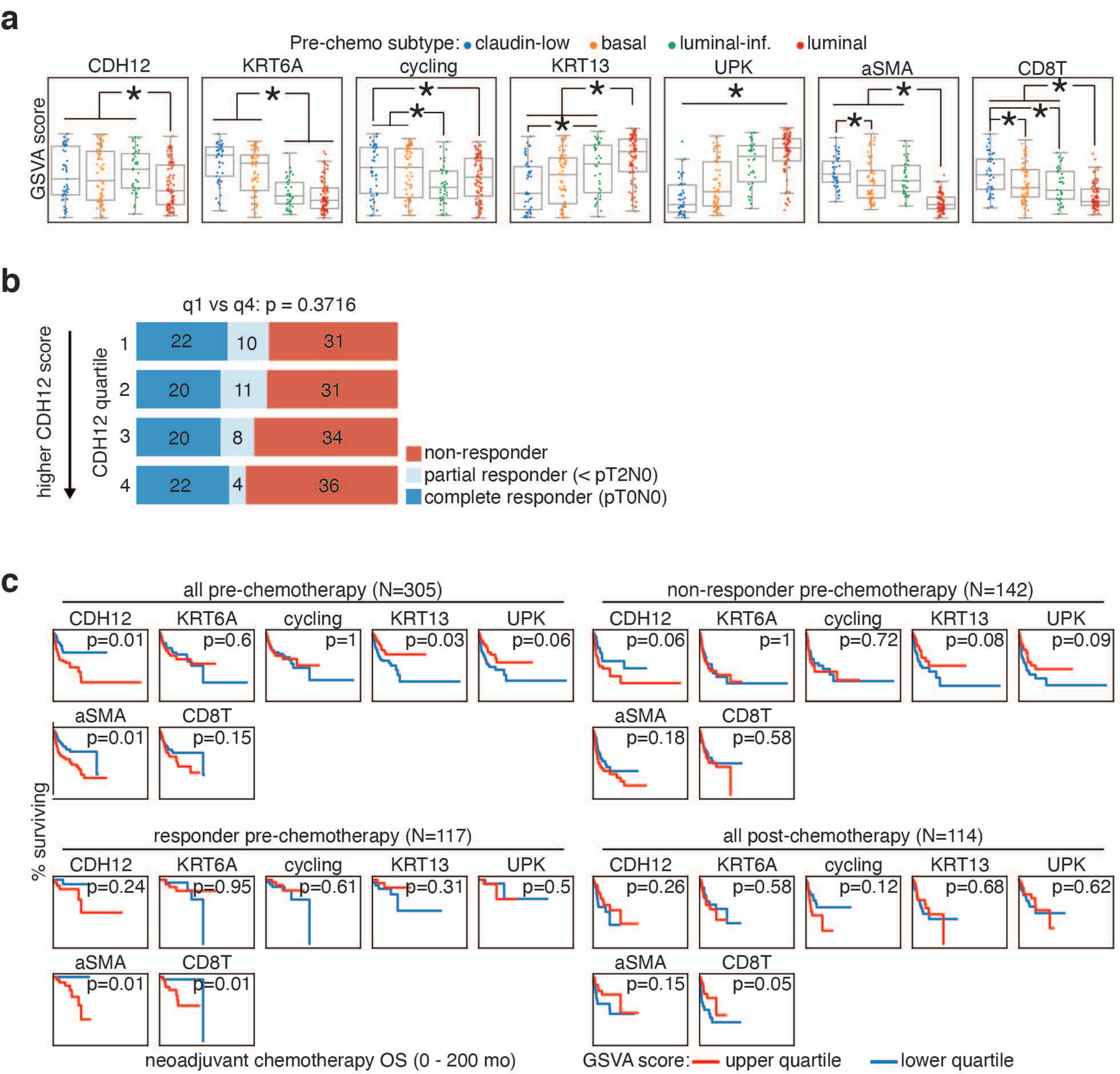

Supplementary Figure 5) snSeq-derived gene signatures in NAC-treated tumors. a, snSeq-derived population signatures in pre-NAC samples per Genomic Subtyping Classifier subtype (n=81 for luminal, n=59 for basal, n=45 for claudin-low, and n=38 for luminal-infiltrated (luminal-inf.) (* indicates two-sided Mann-Whitney p≤0.05. Boxplots are drawn as the inter-quartile range (IQR) with a line indicating the median, and outliers defined as points that fall outside of the range demarcated by 1.5*IQR. **b**, Pathological downstaging of NAC-treated MIBC stratified by pre-NAC CDH12 score quartiles (log-rank test upper versus lower quartiles). **c,** Overall survival in NAC-treated MIBC stratified by snSeq-derived population signatures (log-rank test upper versus lower quartiles). Response was defined as pathologic downstaging (< pT2N0).

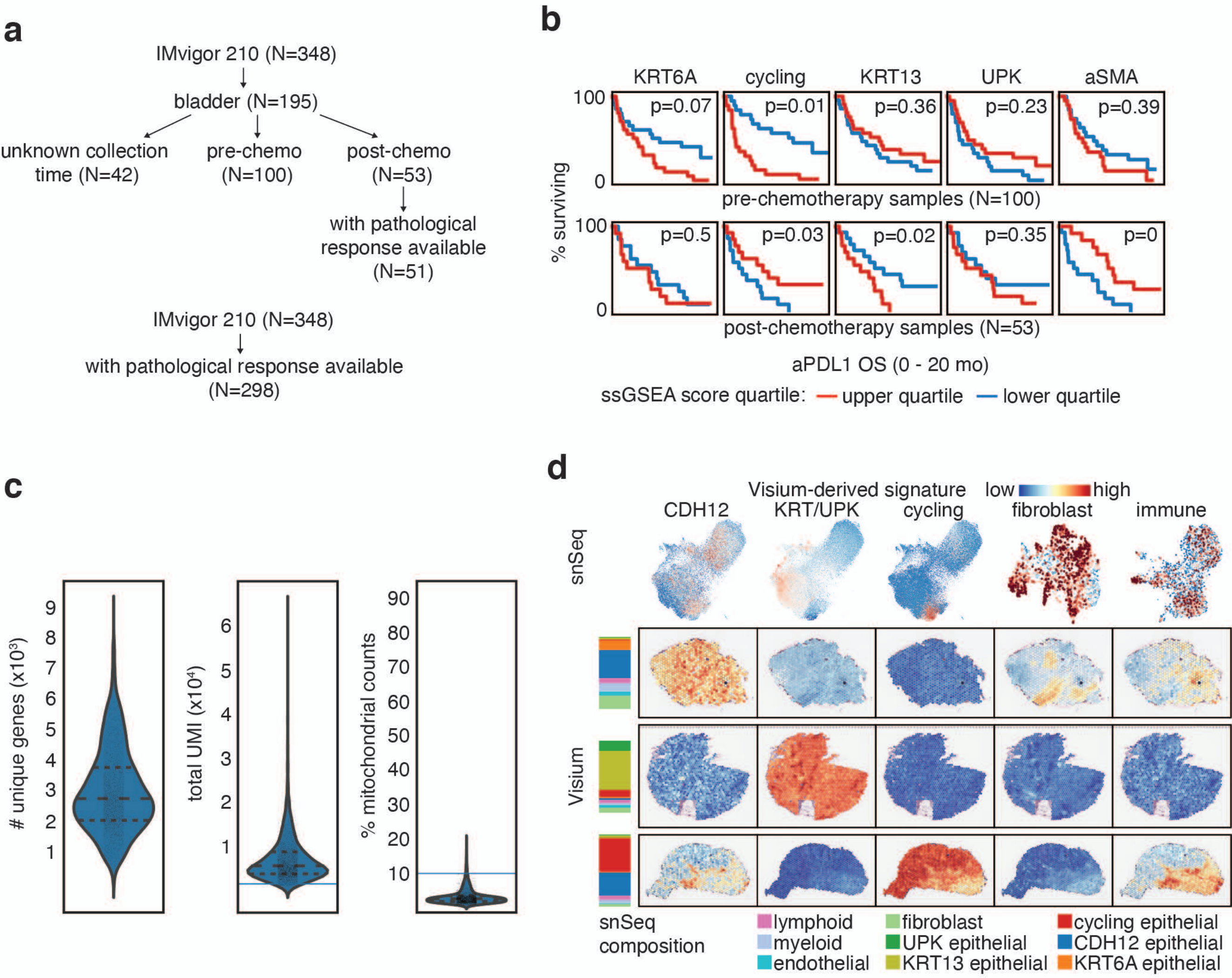

Supplementary Figure 6) Survival prediction in IMvigor 210 by snSeq-derived gene signatures. a, Diagram showing cohort selection for IMvigor 210 analyses. The sample numbers indicate number of samples fitting those criteria for which sequencing data is available. The top diagram shows the selection for the survival analyses and response predictions for all figures except **Main Fig. 6a**. The bottom diagram shows the selection for the response predictions in **Main Fig. 6a**. **b,** Overall survival in IMvigor 210 Cohort 2 bladder tumors sequenced pre-chemo (top, N = 100) or post-chemo (bottom, N = 53) stratified by snSeq-derived population signature scores (log-rank test between top and bottom quartiles; $p = 0$ indicates $p < 0.001$). **c,** QC metrics for Visium dataset where the blue horizontal lines show the cutoffs used for filtering spots. **d**, Visium-derived signature scores in snSeq UMAPs (top) and in-situ on MIBC Visium samples (bottom). Stacked bar plots to the left of each Visium sample show the corresponding snSeq population composition.

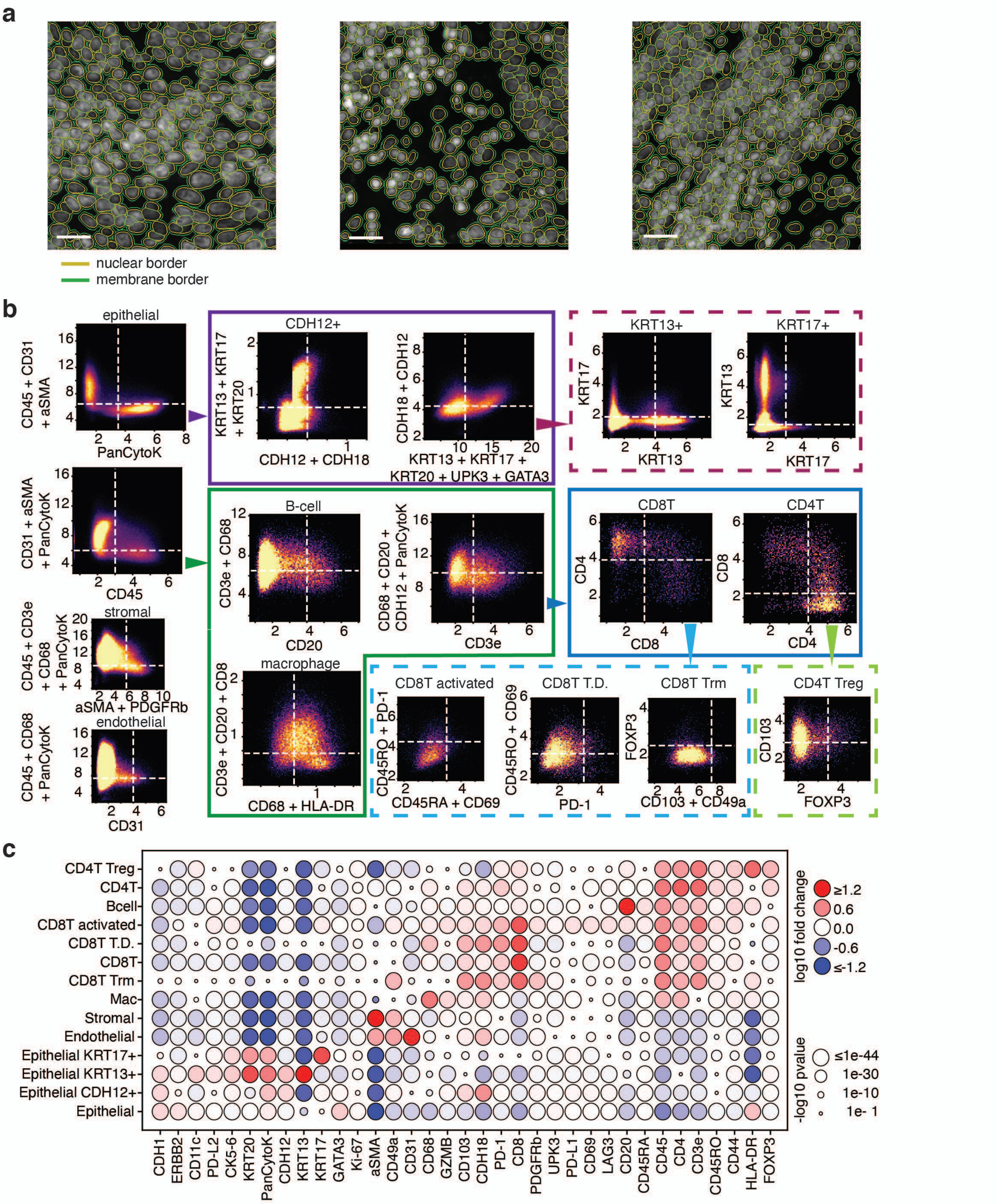

Supplementary Figure 7) CODEX cell type classification and niche identification. a, Example images showing nuclei (DAPI) with nuclear and membrane borders overlaid. Scale bar is 25μm. **b,** CODEX marker intensity gating strategy used to gather training samples for cell subtyping. Cells were partitioned in a hierarchical fashion using combinations of cell lineage markers. When multiple markers are indicated on the same axis, these values were summed together for each cell. Plots outlined in a solid border were used for primary cell typing, and those outlined in a dashed border refer to intensity gates applied to primarily classified cells. **c,** CODEX marker intensity enrichment per cell subtype. Dot hue reflects the log10 fold change, and the size of the dot indicates the Wilcoxon (two-sided) test p-value.

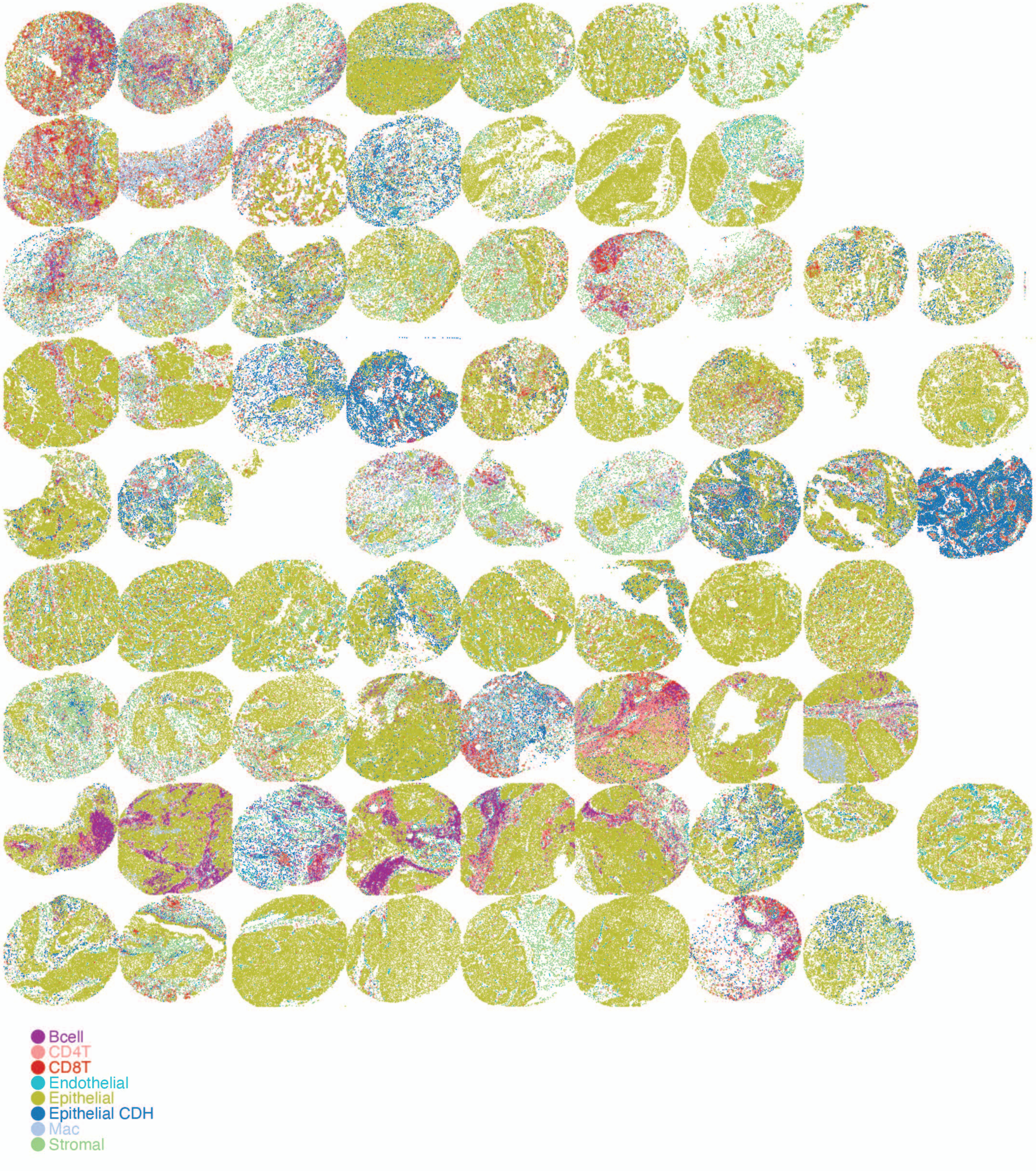

Supplementary Figure 8) CODEX samples annotated by cell type. Every CODEX sample analyzed where each dot represents a cell centroid and is colored by the cell type.

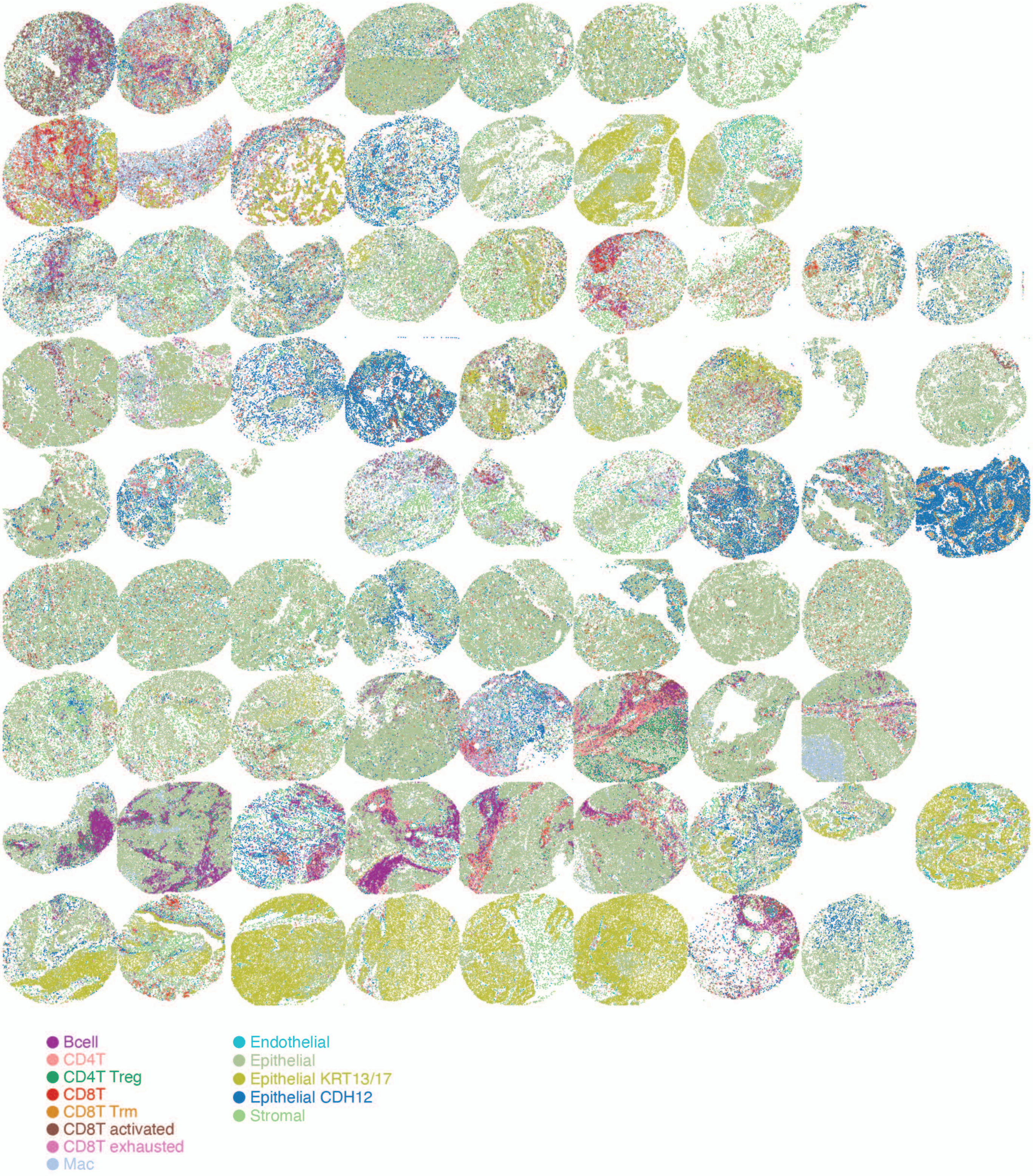

Supplementary Figure 9) CODEX samples annotated by cell subtype. Every CODEX sample analyzed where each dot represents a cell centroid and is colored by the cell subtype.

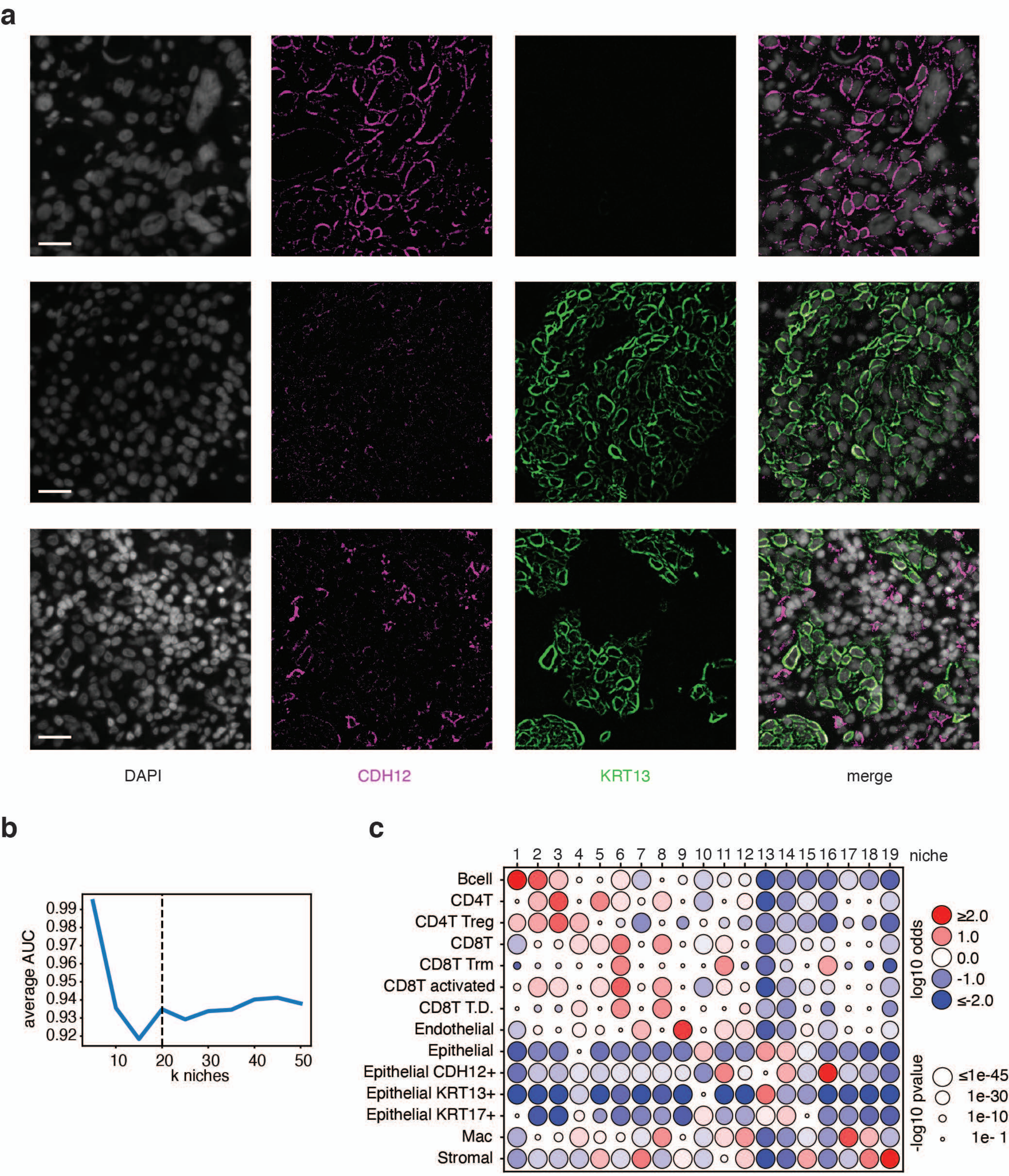

Supplementary Figure 10) CODEX CDH12 and KRT13 staining and derivation of cellular niches (CN). a, Example images showing CDH12 and KRT13 staining on epithelial cells. Scale bar is 25μm. **b,** Average area under the receiver operating characteristic curve (AUC) derived from logistic regression models fit on cellular neighbor profiles (percentage of each broad cell type immediately surrounding each cell) clustered into k clusters. The value of k was varied from 5 to 50 in increments of 5. A high average AUC indicates high predictability of each niche from the others. The vertical dotted line at k=20 indicates the number of cellular niches (CN) chosen for further analysis. **c,** Enrichment of subtypes assigned to each CN compared to any other CN. Dot hue and size reflect Fisher's exact test odds ratio and p-value, respectively.

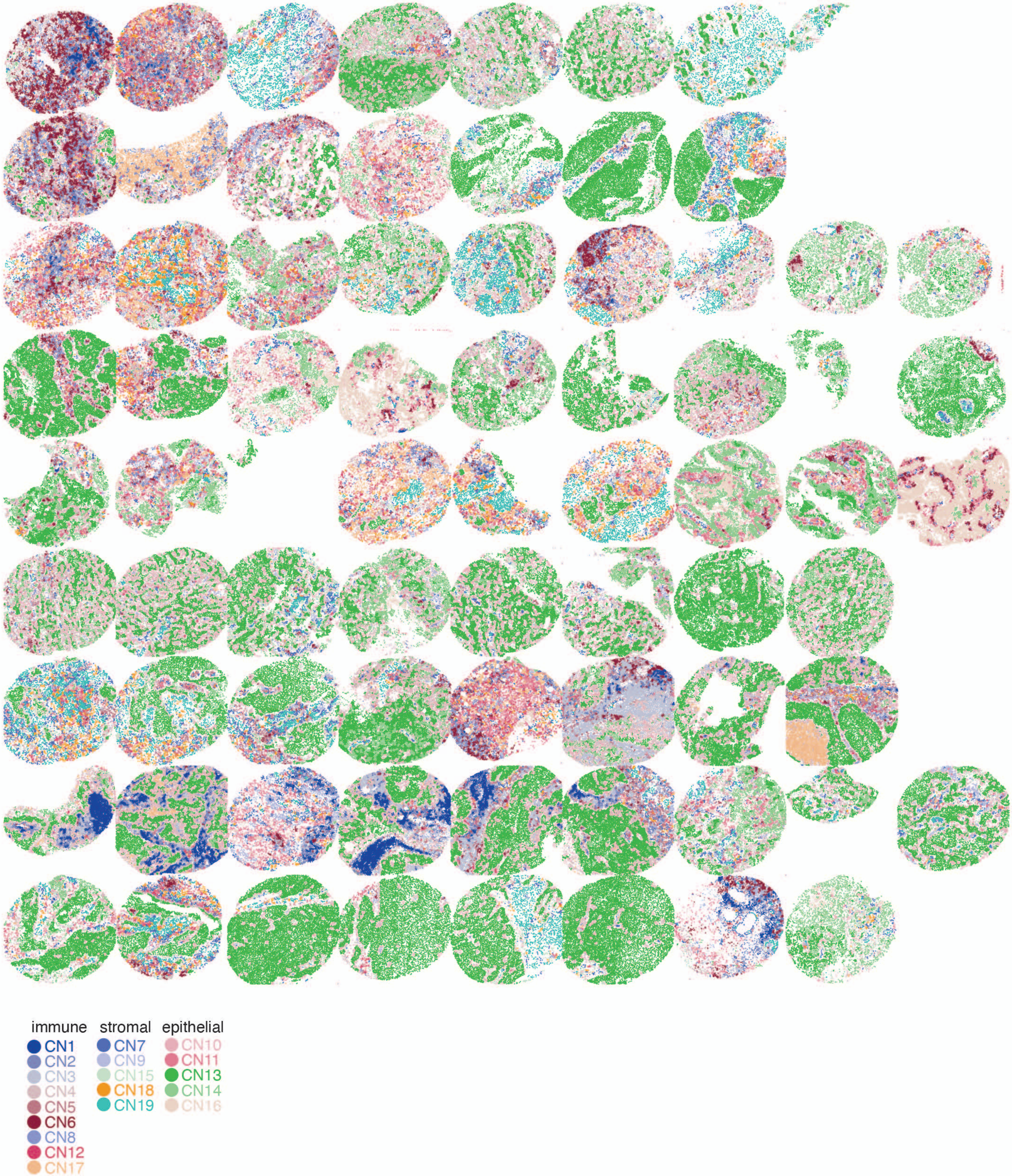

Supplementary Figure 11) CODEX samples annotated by cellular niche (CN). Every CODEX sample analyzed where each dot represents a cell centroid and is colored by the CN to which the cell belongs.

Cohort ID	Grade	Invasive	Recurrence	Tobacco Use	Stage	Dead	Recurrence Days	Outcome
36	High	Yes	No	Yes	T3	0		
54	High	Yes		Yes	T2	0		
72	High	Yes	No	Yes	T2	0	270	
593	High	Yes	No	Yes	T2	1	76	Died
674	High	Yes	Yes	No	T2	0	322	Mets to brain
702	High	Yes	No	Yes	T4	0	40	
739	High	Yes	No	Yes	T2	0	521	
752	High	Yes	No	Yes	T2	0	9	
763	High	Yes	Yes	Yes	T2	0	538	Mets to brain
824	High	No	No	Yes	T1	0		
896	High	Yes	Yes	Yes	T2	0	510	
912	High	Yes	No	No	T2	0	47	Pt refused continued chemo after initial diagnosis
913	High	Yes	No	Yes	T2	0		
1246	High	Yes	Yes	Quit	T2	1		Declined Adj
1126	High	Yes	No	No	T2	0	852	
1204	High	Yes	No	Yes	T2	0	729	
371	High	Yes	No	Yes	T3	0	158	
419	High	Yes	No	Yes	T2	0	54	
446	High	Yes	No	Yes	T2	0	61	
485	High	Yes	No	Yes	T2	0	112	
489	High	Yes	No	Yes	T2	0	1236	
518	High	Yes	No	Yes	T2	0	294	
59	High	Yes	No	Yes	T2	1	178	Dead
590	High	Yes	No	Yes	T2	0	41	
8	High	Yes	No	No	T2	1	150	Dead

Supplementary Table 1 - Patient Clinical Characteristics

Marker	Clone	Manufacturer	Catalog #	Barcode	Fluorophore	Final Dilution	Cycle	Exposure (ms)
CD8	C8/144B	Akoya Biosciences	4250012	BX026	Atto550	400x	2	500
CD4	EPR6855	Akoya Biosciences	4350018	BX003	Cy5	400x	2	350
PanCytoK	AE-1/AE-3	Akoya Biosciences	4450020	BX019	AF750	200x	2	700
aSMA	1A4	Sigma	A5228	BX032	Atto550	400x	3	500
CD45	D9M8I	CST	13917BF	BX021	Cy5	400x	3	350
PDGFRb	Y92	Abcam	ab215978	BX046	AF750	400x	3	700
CD49a	TS2/7	BioLegend	328302	BX002	Atto550	400x	4	500
CD68	KP1	Akoya Biosciences	4350019	BX015	Cy5	400x	4	350
CD31	EP3095	Akoya Biosciences	4450017	BX001	AF750	400x	4	700
CD103	ITGAE/2063	Abcam	ab237854	BX005	Atto550	400x	5	500
HLA-DR	EPR3692	Akoya Biosciences	4450029	BX033	Cy5	200x	5	350
UPK3	EPR14420	Abcam	ab239006	BX025	AF750	50x	5	700
GATA3	L50-823	BD Biosciences	558686	BX014	Atto550	400x	6	500
CD3e	EP449E	Akoya Biosciences	4450030	BX045	Cy5	400x	6	350
Ki-67	SolA15	Thermo-Fisher	14-5698-82	BX049	AF750	100x	6	700
CDH18	6F7	Thermo-Fisher	H00001016-M01	BX029	Atto550	400x	7	500
CDH12	Poly	LSBio	LS-B11408	BX042	Cy5	200x	7	350
KRT13	EPR3671	Abcam	ab239918	BX034	AF750	200x	7	700
KRT17	KRT17/778	Abcam	ab212553	BX020	Atto550	400x	8	500
CK5/6	D5/16B4	Sigma	MAB1620	BX006	Cy5	400x	8	350
CDH1	4A2C7	Thermo-Fisher	33-4000	BX028	AF750	50x	8	700
PD-1	NAT105	Abcam	ab201811	BX023	Atto550	400x	9	500
CD11c	118/A5	Akoya Biosciences	4350020	BX024	Cy5	400x	9	350
KRT20	2G3-1C8	LSBio	LS-B4527	BX022	AF750	400x	9	700
CD69	EPR21814	Abcam	ab234512	BX017	Atto550	400x	10	500
PD-L2	D7U8C	CST	82723BF	BX027	Cy5	200x	10	350
CD20	L26	Akoya Biosciences	4450018	BX007	AF750	400x	10	700
PD-L1	28-8	Abcam	ab209889	BX047	Atto550	400x	11	500
FOXP3	PCH101	Thermo-Fisher	14-4776-82	BX030	Cy5	400x	11	350
ERBB2	3B5	Abcam	ab16901	BX031	AF750	200x	11	700
GZMB	496B	Thermo-Fisher	14-8889-82	BX035	Atto550	400x	12	500
CD44	G44-26 (C26)	BD Biosciences	555476	BX036	Cy5	400x	12	350
CD45RO	UCHL1	BioLegend	304202	BX040	AF750	100x	12	700
LAG3	D2G40	CST	15372BF	BX041	Atto550	400x	13	500
CD45RA	HI100	BioLegend	304102	BX004	AF750	100x	13	700

Supplementary Table 2 - CODEX antibodies details
