# Supplementary material for: An N-Cadherin 2 expressing epithelial cell subpopulation predicts response to surgery, chemotherapy and immunotherapy in bladder cancer
Source: Nat Commun. 2021 Aug 12;12:4906. doi: 10.1038/s41467-021-25103-7 (PMC8361097; doi:10.1038/s41467-021-25103-7)
Supplement: Supplementary file 2 — Description of Additional Supplementary File [file 41467_2021_25103_MOESM2_ESM.pdf]

Description of Additional Supplementary Files

Supplementary Data 1: Complete differential gene expression analysis results for MIBC epithelial clusters

Supplementary Data 2: TCGA samples used (N=259)

Supplementary Data 3: Gene signatures used for ssGSEA analysis of bulk RNA-seq data
