# Supplementary material for: An N-Cadherin 2 expressing epithelial cell subpopulation predicts response to surgery, chemotherapy and immunotherapy in bladder cancer
Source: Nat Commun. 2021 Aug 12;12:4906. doi: 10.1038/s41467-021-25103-7 (PMC8361097; doi:10.1038/s41467-021-25103-7)
Supplement: Supplementary file 6 — Reporting Summary [file 41467_2021_25103_MOESM6_ESM.pdf]

nature research

Corresponding author(s): Dan Theodorescu and Simon R.V. Knott

Last updated by author(s): Jul 1, 2021

Reporting Summary

Nature Research wishes to improve the reproducibility of the work that we publish. This form provides structure for consistency and transparency in reporting. For further information on Nature Research policies, see our Editorial Policies and the Editorial Policy Checklist.

Statistics

For all statistical analyses, confirm that the following items are present in the figure legend, table legend, main text, or Methods section.

n/a	Confirmed	
☐	☒	The exact sample size (*n*) for each experimental group/condition, given as a discrete number and unit of measurement
☐	☒	A statement on whether measurements were taken from distinct samples or whether the same sample was measured repeatedly
☐	☒	The statistical test(s) used AND whether they are one- or two-sided<br>*Only common tests should be described solely by name; describe more complex techniques in the Methods section.*
☒	☐	A description of all covariates tested
☐	☒	A description of any assumptions or corrections, such as tests of normality and adjustment for multiple comparisons
☐	☒	A full description of the statistical parameters including central tendency (e.g. means) or other basic estimates (e.g. regression coefficient) AND variation (e.g. standard deviation) or associated estimates of uncertainty (e.g. confidence intervals)
☐	☒	For null hypothesis testing, the test statistic (e.g. *F*, *t*, *r*) with confidence intervals, effect sizes, degrees of freedom and *P* value noted<br>*Give P values as exact values whenever suitable.*
☒	☐	For Bayesian analysis, information on the choice of priors and Markov chain Monte Carlo settings
☒	☐	For hierarchical and complex designs, identification of the appropriate level for tests and full reporting of outcomes
☒	☐	Estimates of effect sizes (e.g. Cohen's *d*, Pearson's *r*), indicating how they were calculated

Our web collection on statistics for biologists contains articles on many of the points above.

Software and code

Policy information about availability of computer code

Data collection	Data from the IMvigor210 trial were obtained from the IMvigor210CoreBiologies R package, made freely available by the authors of the trial manuscript.
Data analysis	Data analysis was performed using standard packages in python or R as listed below:<br>CellRanger (10X genomics, v3.0.2)<br>Seurat (v3.2.3)<br>Cite-seq-count (v1.4.3)<br>Scrublet (v0.2.1)<br>scanpy v1.5.1<br>SpaceRanger (10X genomics, v1.1.0)<br>oligo (v1.52.1)<br>scVI v0.6.8<br>Rapids.ai cuml v0.12.0<br>cugraph v0.17<br>pySCENIC v0.10.0<br>scVelo v0.17.15<br>GSEApy v0.10.1<br>GSVA v1.36.2<br>lifelines v0.25.4<br>limma (v3.44.3)<br>SpatialDE (v1.1.3)<br>scipy v1.4.1

cuML v0.17
opencv v3.4.2

Custom analysis pipelines were implemented in Matlab, R, or python and are available at https://github.com/KnottLab/bladder-snSeq and https://github.com/KnottLab/codex.

For manuscripts utilizing custom algorithms or software that are central to the research but not yet described in published literature, software must be made available to editors and reviewers. We strongly encourage code deposition in a community repository (e.g. GitHub). See the Nature Research guidelines for submitting code & software for further information.

Data

Policy information about availability of data

All manuscripts must include a data availability statement. This statement should provide the following information, where applicable:

- Accession codes, unique identifiers, or web links for publicly available datasets
- A list of figures that have associated raw data
- A description of any restrictions on data availability

The following datasets were generated in this study. Single-nuclei RNA-seq and HTO data have been deposited in the GEO database under accession code GSE169379. Visium data have been deposited in the GEO database under accession code GSE171351. CODEX processed data are available through figshare from the following links: https://figshare.com/s/4610a15363c8306dfa36, https://figshare.com/s/2005255a8b65de23109f, https://figshare.com/s/1d8c7ed76d4b3222ada4. CODEX raw data are available from the corresponding authors upon reasonable request.

The following datasets are publicly available. Bladder urothelial carcinoma Illumina Hi-Seq counts from The Cancer Genome Atlas (TCGA) were downloaded from the Genomic Data Commons (GDC) data portal, and corresponding clinical annotation including survival information was accessed via the TCGA Clinical Data Resource. Data from the IMvigor210 trial were obtained from the IMvigor210CoreBiologies R package, made freely available by the authors of the trial manuscript. Affymetrix array data corresponding to a trial of neoadjuvant cisplatin-based chemotherapy in MIBC was downloaded from GEO (GSE124305 and GSE87304).

The remaining data are available within the Article, Supplementary Information or Source Data file.

Field-specific reporting

Please select the one below that is the best fit for your research. If you are not sure, read the appropriate sections before making your selection.

- [x] Life sciences
- [ ] Behavioural & social sciences
- [ ] Ecological, evolutionary & environmental sciences

For a reference copy of the document with all sections, see nature.com/documents/nr-reporting-summary-flat.pdf

Life sciences study design

All studies must disclose on these points even when the disclosure is negative.

Sample size	Sample size was constrained by the material available for biobanked tissues.
Data exclusions	Data were only excluded on the basis of standard quality control metrics for single-cell RNA-sequencing methods, which are described in detail in the Methods section.
Replication	This is a dataset generated from biobanked, archival patient material, and thus cannot be replicated per se; however, relevant papers supporting the clinical findings were cited in our manuscript. Our general findings can be reproduced using our reported methodology, however our specific samples cannot be replicated because they are patient material.
Randomization	Randomization was not used in this study as the data were generated as a descriptive dataset. All of the samples were combined into a comprehensive analysis, and we are not making any conclusions based on comparisons of samples within our dataset, therefore randomization is not necessary.
Blinding	Samples were collected by the physician, and therefore there was no blinding at that stage of the study. Samples were processed by researchers who only had access to de-identified patient IDs, and therefore the researchers were blinded to any clinical characteristics associated with the samples. De-identified patient IDs were also used for the computational analysis. All analysis was performed computationally, and therefore there is no subjective analysis included in the results of this study.

Reporting for specific materials, systems and methods

We require information from authors about some types of materials, experimental systems and methods used in many studies. Here, indicate whether each material, system or method listed is relevant to your study. If you are not sure if a list item applies to your research, read the appropriate section before selecting a response.

Materials & experimental systems

n/a	Involved in the study
☐	☒ Antibodies
☒	☐ Eukaryotic cell lines
☒	☐ Palaeontology and archaeology
☒	☐ Animals and other organisms
☐	☒ Human research participants
☒	☐ Clinical data
☒	☐ Dual use research of concern

Methods

n/a	Involved in the study
☒	☐ ChIP-seq
☒	☐ Flow cytometry
☒	☐ MRI-based neuroimaging

Antibodies

Antibodies used

TotalSeq-A 0451 anti-Nuclear Hashtag 1 (Biolegend, custom formulation provided by Biolegend, clone MAB414, 1.5ug per sample)
TotalSeq-A 0452 anti-Nuclear Hashtag 2 (Biolegend, custom formulation provided by Biolegend, clone MAB414, 1.5ug per sample)
TotalSeq-A 0453 anti-Nuclear Hashtag 3 (Biolegend, custom formulation provided by Biolegend, clone MAB414, 1.5ug per sample)
TotalSeq-A 0454 anti-Nuclear Hashtag 4 (Biolegend, custom formulation provided by Biolegend, clone MAB414, 1.5ug per sample)
TotalSeq-A 0455 anti-Nuclear Hashtag 5 (Biolegend, custom formulation provided by Biolegend, clone MAB414, 1.5ug per sample)
TotalSeq-A 0456 anti-Nuclear Hashtag 6 (Biolegend, custom formulation provided by Biolegend, clone MAB414, 1.5ug per sample)
KRT13 (Abcam, cat: ab239918, clone EPR3671, 1:100)
KRT17 (Abcam, cat: ab212553, clone KRT17/778, 1:100)
CDH12 (LSBio, cat: LS-B11408-100, rabbit polyclonal, 1:100)
CDH18 (Thermo-Fisher Scientific, cat: H00001016-M01, clone 6F7, 1:50)
CD8 (Akoya Biosciences, cat: 4250012, clone: C8/144B, 1:400)
CD4 (Akoya Biosciences, cat: 4350018, clone: EPR6855, 1:400)
PanCytoK (Akoya Biosciences, cat: 4450020, clone: AE-1/AE-3, 1:200)
aSMA (Millipore-Sigma, cat: A5228, clone: 1A4, 1:400)
CD45 (CST, cat: 13917BF, clone: D9M8I, 1:400)
PDGFRb (Abcam, cat: ab215978, clone: Y92, 1:400)
CD49a (BioLegend, cat: 328302, clone: TS2/7, 1:400)
CD68 (Akoya Biosciences, cat: 4350019, clone: KP1, 1:400)
CD31 (Akoya Biosciences, cat: 4450017, clone: EP3095, 1:400)
CD103 (Abcam, cat: ab237854, clone: ITGAE/2063, 1:400)
HLA-DR (Akoya Biosciences, cat: 4450029, clone: EPR3692, 1:200)
UPK3 (Abcam, cat: ab239006, clone: EPR14420, 1:50)
GATA3 (BD Biosciences, cat: 558686, clone: L50-823, 1:400)
CD3e (Akoya Biosciences, cat: 4450030, clone: EP449E, 1:400)
Ki-67 (Thermo-Fisher, cat: 14-5698-82, clone: SolA15, 1:100)
CK5/6 (Millipore-Sigma cat: MAB1620, clone: D5/16B4, 1:400)
CDH1 (Thermo-Fisher, cat: 33-4000, clone: 4A2C7, 1:50)
PD-1 (Abcam, cat: ab201811, clone: NAT105, 1:400)
CD11c (Akoya Biosciences, cat: 4350020, clone: 118/A5, 1:400)
KRT20 (LSBio, cat: LS-B4527, clone: 2G3-1C8, 1:400)
CD69 (Abcam, cat: ab234512, clone: EPR21814, 1:400)
PD-L2 (CST, cat: 82723BF, clone: D7U8C, 1:200)
CD20 (Akoya Biosciences, cat: 4450018, clone: L26, 1:400)
PD-L1 (Abcam, cat: ab209889, clone: 28-8, 1:400)
FOXP3 (Thermo-Fisher, cat: 14-4776-82, clone: PCH101, 1:400)
ERBB2 (Abcam, cat: ab16901, clone: 3B5, 1:200)
GZMB (Thermo-Fisher, cat: 14-8889-82, clone: 496B, 1:400)
CD44 (BD Biosciences, cat: 555476, clone: G44-26 (C26), 1:400)
CD45RO (BioLegend, cat: 304202, clone: UCHL1, 1:100)
LAG3 (CST, cat: 15372BF, clone: D2G40, 1:400)
CD45RA (BioLegend, cat: 304102, clone: HI100, 1:100)
donkey anti-mouse IgG AF568 (Thermo Fisher Scientific, cat: A10037, 1:500)
goat anti-rabbit IgG AF488 (Thermo Fisher Scientific, cat: A11008, 1:500)

Validation

Validation is provided on the manufacturer's website for each antibody. Antibodies were titrated in our own hands using appropriate positive and negative control tissue to establish specificity.

Human research participants

Policy information about studies involving human research participants

Population characteristics	Complete population characteristics are reported in Supplementary Table 1.
Recruitment	All potentially eligible subjects for our tumor bank were approached in order to minimize the possibility that the study population is not representative of the clinical population seen at the study site.
Ethics oversight	The Research Ethics Committee of Cedars-Sinai Medical Center approved the study (Study00000542).

Note that full information on the approval of the study protocol must also be provided in the manuscript.
